# Therapeutic Hypothermia Modulates the Relationships Between Indicators of Severity of Neonatal Hypoxic Ischemic Encephalopathy and Serum Biomarkers

**DOI:** 10.3389/fneur.2021.748150

**Published:** 2021-11-02

**Authors:** Raul Chavez-Valdez, Sarah Miller, Harisa Spahic, Dhananjay Vaidya, Charlamaine Parkinson, Barbara Dietrick, Sandra Brooks, Gwendolyn J. Gerner, Aylin Tekes, Ernest M. Graham, Frances J. Northington, Allen D. Everett

**Affiliations:** ^1^Department of Pediatrics, Division of Neonatology, Johns Hopkins University School of Medicine, Baltimore, MD, United States; ^2^Neuroscience Intensive Care Nursery Program, Division of Neonatology, Johns Hopkins University School of Medicine, Baltimore, MD, United States; ^3^Department of Internal Medicine, Johns Hopkins University School of Medicine, Baltimore, MD, United States; ^4^Department of Pediatrics, Division of Neonatology, Johns Hopkins All Children's Hospital, St Petersburg, FL, United States; ^5^Department of Neuropsychology, Kennedy Krieger Institute, Baltimore, MD, United States; ^6^Department of Psychiatry and Behavioral Sciences, Johns Hopkins University School of Medicine, Baltimore, MD, United States; ^7^Department of Radiology, Division of Pediatric Radiology and Pediatric Neuroradiology, Johns Hopkins Hospital, Baltimore, MD, United States; ^8^Department of Obstetrics and Gynecology, Johns Hopkins University School of Medicine, Baltimore, MD, United States; ^9^Department of Pediatrics, Division of Pediatric Cardiology, Johns Hopkins University School of Medicine, Baltimore, MD, United States

**Keywords:** neonatal encephalopathy, cytokines, neurotrophins, GFAP, tau, Sarnat score

## Abstract

**Objective:** To determine the changes due to therapeutic hypothermia (TH) exposure in the strength of association between traditional clinical and biochemical indicators of severity of neonatal hypoxic-ischemic encephalopathy (HIE) and serum biomarkers. We hypothesized that culmination of TH changes the strength of the relationships between traditional indicators of severity of HIE and serum biomarkers.

**Methods:** This was a single-center observational cohort study of 178 neonates with HIE treated with TH and followed with serum biomarkers: (i) brain-derived neurotrophic factor (BDNF) and vascular endothelial growth factor (VEGF) (neurotrophins); (ii) tau and glial fibrillary acidic protein (GFAP) (neural cell injury); and (iii) interleukin 6 (IL-6), IL-8, and IL-10 (cytokines), during their first week of life. Adjusted mixed-effect models tested associations with HIE indicators in relation to TH exposure.

**Results:** At admission, lower Apgar scores and base excess (BE) and higher lactate and nucleated red blood cell (NRBC) count correlated with higher Sarnat scores. These indicators of worse HIE severity, including higher Sarnat score, correlated with lower VEGF and higher tau, GFAP, and IL-10 levels at different time points. Within the first 24 h of life, patients with a Sarnat score >2 had lower VEGF levels, whereas only those with score of 3 also had higher GFAP and IL-10 levels. Tau levels increased during TH in patients with Sarnat score of 3, whereas tau and GFAP increased after TH in those with scores of 2. After adjustments, lower VEGF levels during TH and higher tau, GFAP, and IL-10 levels during and after TH were associated with worse Sarnat scores. Tau and GFAP relationship with Sarnat score became stronger after TH.

**Conclusion:** Therapeutic hypothermia exerts an independent modulatory effect in the relationships between traditional indicators of severity of HIE and serum biomarkers after adjustments. Thus, the timing of biomarker testing in relation to TH exposure must be carefully considered if biomarkers are proposed for patient stratification in novel clinical trials.

## Introduction

Hypoxic-ischemic (HI) encephalopathy (HIE) is the most prevalent type of brain injury in full-term neonates and a main cause of neonatal encephalopathy (NE) (1). Impaired fetal perfusion results in biochemical changes, such as worsening acidosis and ultimately HI injury to the brain (1, 2). Hypoxic-ischemic encephalopathy results from a cascade of excitotoxicity, oxidative stress, and inflammation, which may persist for weeks (3–5). The severity and recovery of HI brain injury do not always align with early assessments using traditional clinical and biochemical indicators because of lack of sensitivity and specificity to brain injury. Therapeutic hypothermia (TH), the only available therapy, reduces death or disability in patients with moderate HIE (6–9). However, sex and other factors (5, 10–13) may play a role in the variable post-natal presentation and response to TH. The lack of specificity of the existing criteria guiding the initiation of TH offers an unsatisfactory assessment of severity of brain injury hindering our ability to identify patients who may benefit of adjuvant therapeutic strategies and to monitor response to therapy (1, 14). Thus, peripheral blood biomarkers may improve the assessment provided by traditional indicators alone.

Brain insults trigger activation of signaling cascades, resulting in a temporally dynamic “biochemical footprint” detectable in circulating blood that have the potential to be used as biomarkers. These biomarkers may improve the ability to grade severity of injury and guide therapy in several neurological conditions (15–17). In neonatal HIE, brain-derived neurotrophic factor (BDNF), glial fibrillary acidic protein (GFAP), and tau have been among those studied against modified Sarnat scores, as well as brain magnetic resonance imaging (MRI) and neurodevelopmental outcomes (18, 19). However, it is unclear how longitudinal changes in these and other proposed HIE biomarkers relate to traditional early clinical and biochemical indicators in the era of TH. Understanding these relationships and how TH exposure may modulate them will inform the optimal timing at which proposed biomarkers may indicate the specific type and severity of HI injury to guide treatment and refine predictive models.

Here, we aim to study the changes attributable to TH exposure in the strength of association between traditional clinical and biochemical indicators of severity of neonatal HIE and serum biomarkers. These biomarkers included (i) BDNF and vascular endothelial growth factor (VEGF), neurotrophins essential for brain development (18, 20, 21); (ii) tau and GFAP, neuronal (19, 22, 23), and astrocytic (24) cytoskeletal markers linked to brain cell injury; and (iii) interleukin 6 (IL-6), IL-8, and IL-10, markers of inflammatory response (18, 20, 21); most of them studied in association to neonatal HIE previously in smaller cohorts. We hypothesized that culmination of TH changes the strength of the relationship between traditional indicators of severity of HIE and serum biomarkers. Here, we report that (i) indicators of worse HIE severity, including higher Sarnat score, correlated with lower VEGF and higher tau, GFAP, and IL-10 levels; and (ii) lower VEGF levels during TH, and higher tau, GFAP, and IL-10 levels during and after TH, were associated with worse Sarnat scores with tau and GFAP relationship with Sarnat score becoming stronger after TH.

## Methods

The study received exempt status from the Johns Hopkins University (JHU) institutional review board (IRB), until 2017, when informed consent became required to access medical records and use discarded blood collected for clinical purposes. The Neuroscience Intensive Care Nursery program coordinator (C.P.) identified patients with diagnosis of NE and obtained agreement by the treating clinical team to allow a member of the study team to discuss inclusion of the neonate in the study with the parents. Written consent was obtained from the parents of the participants after 2017, as required by IRB. The study Identification of Diagnostic and Prognostic Biomarkers for Perinatal Hypoxic-Ischemic Brain Injury (BIN study, IRB NA_00026068) was in compliance with the Health Insurance Portability and Accountability Act of 1996 (HIPAA).

### Patients

#### Inclusion Criteria

A prospective cohort of 246 neonates (GA ≥35 weeks) diagnosed with NE, who were initiated in whole-body TH within 6 h of life between April 28, 2009, and November 15, 2019, at a single level IV neonatal intensive care unit (NICU).

#### Exclusion Criteria

**Figure 1:**
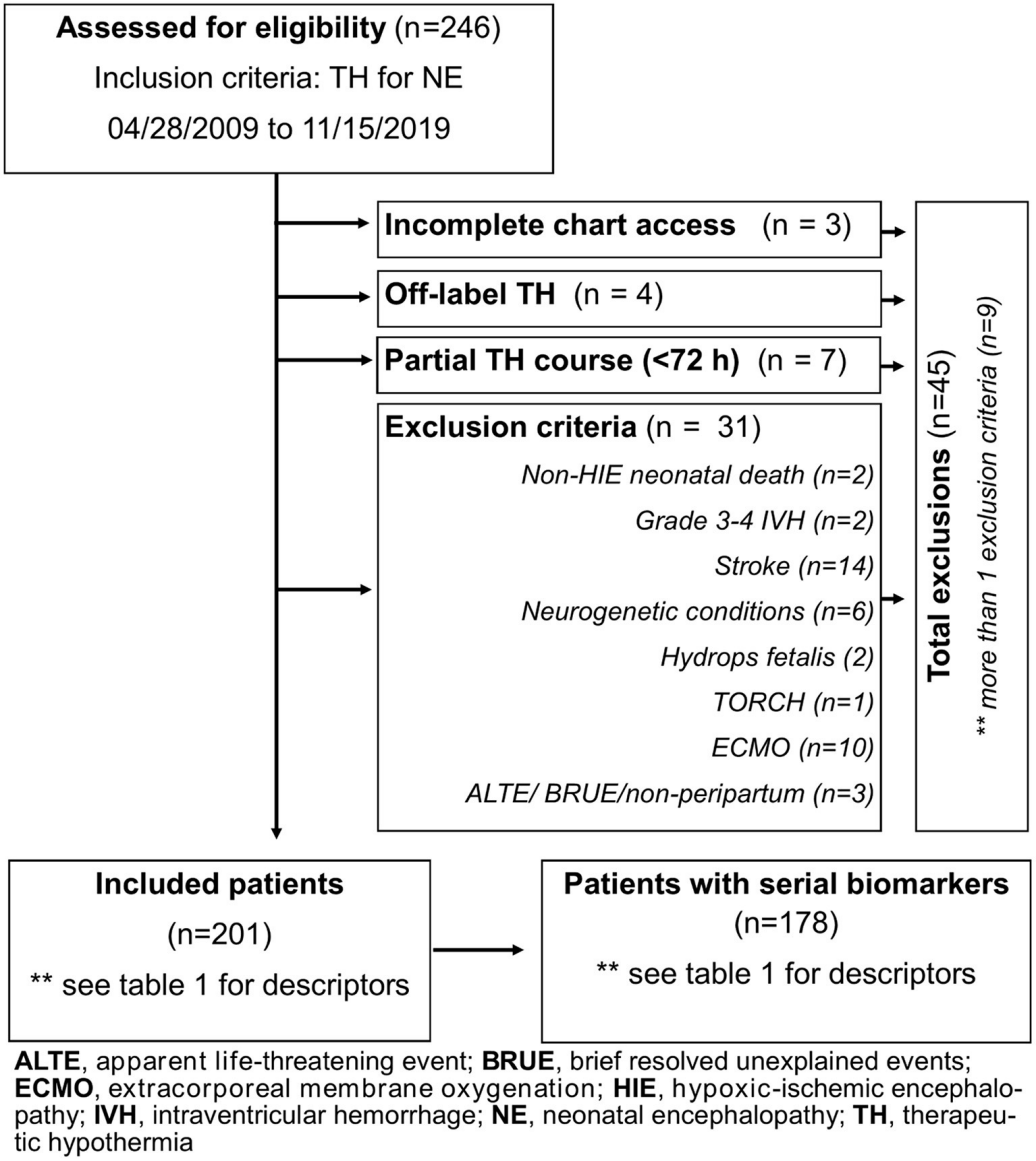
Flowchart of patient allocation.

Forty-five patients were excluded for incomplete clinical data, off-label use of TH (<35 weeks GA), partial TH course, non-peripartum events (apparent life-threatening event/brief, resolved, unexplained event), death for causes other than HIE (i.e., severe pulmonary hypertension of the newborn), need for extracorporeal membrane oxygenation, and non-HIE causes of NE (Figure 1). Of 201 patients, 178 had 7 days of biomarker data. Diagnosis of HIE was based on the National Institute of Child Health and Human Development (NICHD) Neonatal Research Network criteria (6).

#### Whole-Body TH Protocol

Initiation of TH occurred within the first 6 h of life in infants ≥1,800 g birth weight and ≥35 weeks GA with (a) cord or first hour of life pH ≤ 7.0 and/or base excess (BE) ≤-16 or (b) cord or first hour pH > 7.0 but ≤7.15 and/or BE between −10 and −16, plus evidence of a perinatal adverse event and need for assisted ventilation or Apgar <5 at 10 min of life. Although moderate to severe encephalopathy is a criterion used to initiate TH, ~20% of patients included in the analysis had a modified Sarnat score (25, 26) of 1 due to concerns that the neurological examination may worsen after 6 h of life (27–29), particularly in those with severe metabolic acidosis (30, 31). In our cohort, 83% of patients with Sarnat score of 1 who received TH had severe metabolic acidosis. The inclusion of these patients in the analysis better reflects the current state of practice.

### Clinical Data

Clinical data were obtained from electronic charts (Table 1). Race was assigned based on maternal race. Absent/reversed end-diastolic flow in umbilical artery was determined by Doppler scan of the umbilical artery. Placenta pathology was used to assign histological chorioamnionitis or funisitis. Assignment of sex of the newborn, confirmation of GA, and Apgar scores were determined by the NICU team during initial assessment. Highest modified Sarnat score during the first 6 h of admission to the NICU was determined by the study team (R.C.V., C.P., and F.J.N.) (25, 26). Most deaths occurring in the cohort were secondary to withdrawal of care in conjunction with the family after discussion about clinical prognosis.

**Table 1:** Summary of prenatal, perinatal, and postnatal patient variables.

**PRENATAL**	**All HIE patients**	**With biomarker data**	**Without biomarker data**	***p*-value**
	**(*n* = 201)**	**(*n* = 178)**	**(*n* = 23)**	
**Maternal age**, median (IQR), *n*	29 (25, 33), 201	29 (25, 33), 178	29.0 (24.0, 32.0), 23	0.80^a^
**Gravida**, median (IQR), *n*	2 (1,3), 201	2 (1,3), 178	2.0 (1.0, 5.0), 23	0.50^a^
**Race**, black, *n* (%)	76 (38.0%)	68 (38.4%)	8 (34.8%)	0.86^b^
**GA** (weeks), median (IQR), *n*	39.1 (37.7, 40.1), 201	39.1 (37.6, 40.1), 178	39.3 (37.9, 40.1), 23	0.81^a^
**Sex**, Male, *n* (%)	115 (57.2%)	100 (56.2%)	15 (65.2%)	0.41^b^
**Preeclampsia**, *n* (%)	24 (11.9%)	19 (10.7%)	5 (21.7%)	0.12^b^
**IUGR**, *n* (%)	8 (4.0%)	6 (3.4%)	2 (8.7%)	0.22^b^
**Oligohydramnios**, *n* (%)	5 (2.5%)	4 (2.2%)	1 (4.3%)	0.54^b^
**Tobacco exposure**, *n* (%)	9 (4.5%)	8 (4.5%)	1 (4.3%)	0.97^b^
**Opioid exposure**, *n* (%)	4 (2.0%)	2 (1.1%)	2 (8.7%)	***0.02*** ^b^
**Cocaine exposure**, *n* (%)	2 (1.0%)	2 (1.1%)	0 (0.0%)	0.61^b^
**PERINATAL**				
**BW** (g), median (IQR), *n*	3,255 (2,900, 3,680), 201	3,235 (2,885, 3,670),178	3,371 (2,960, 4,085), 23	0.26^a^
**Delivery**, C/S, *n* (%)	137 (68.2%)	120 (67.4%)	17 (73.9%)	0.81^b^
**Emergent C/S**, *n* (%)	119 (60.1%)	105 (60.0%)	14 (60.9%)	0.94^b^
**NRFHT**, *n* (%)	120 (59.7%)	105 (59.0%)	15 (65.2%)	0.57^b^
**Placental abruption**, *n* (%)	35 (17.4%)	31 (17.4%)	4 (17.4%)	1.00^b^
**Clinical chorio**, *n* (%)	23 (11.4%)	22 (12.4%)	1 (4.3%)	0.26^b^
**Chorio (pathology)**, *n* (%)	28 (36.0%)	26 (37.0%)	2 (29.0%)	0.65^b^
**Funisitis (pathology)**, *n* (%)	14 (18.0%)	12 (17.0%)	2 (22.0%)	0.71^b^
**Labor induction**, *n* (%)	68 (33.8%)	64 (36.0%)	4 (17.4%)	*0.08* ^b^
**Sentinel event**, *n* (%)	80 (39.8%)	71 (39.9%)	9 (39.1%)	0.94^b^
**Meconium-stained AF**, *n* (%)	71 (35.3%)	58 (32.6%)	13 (56.5%)	***0.02*** ^b^
**POSTNATAL**				
**Apgar 1 m**, median (IQR), *n*	1 (1,2), 191	1 (1,2), 172	1.0 (0.0, 1.0), 19	***0.03*** ^a^
**Apgar 5 m**, median (IQR), *n*	4 (3,5), 191	4 (3,5), 172	3.0 (0.0, 6.0), 19	0.15^a^
**Apgar 10 m**, median (IQR), *n*	6 (4,7), 166	6 (4,7), 148	4.5 (3.0, 7.0), 18	0.43^a^
**Worse pH**, median (IQR), *n*	6.95 (6.85, 7.07), 201	6.95 (6.85, 7.07), 178	6.96 (6.80, 7.04), 23	0.75^a^
**Worse BE**, median (IQR), *n*	−17.0 (−22, −13.9), 200	−16.3 (−21, −14), 177	−18.0 (−25.0, −13.0), 23	0.24^a^
**Lactate (mmol/L)**, median (IQR), *n*	5.5 (2.6, 9.8), 125	5.1 (2.5, 9.5), 109	7.1 (3.5, 11.0), 16	0.14^a^
**Hct (%)**, median (IQR), *n*	46 (40.8, 50.8), 201	45.6 (40.6, 51.0), 178	46.4 (42.8, 49.1), 23	0.63^a^
**NRBCs/mm**^**3**^, median (IQR), *n*	1,650 (430, 4,350), 199	1,580 (420, 4,180), 176	2,460 (1,270, 6,780), 23	0.24^a^
**Sarnat score**, median (IQR), *n*	2 (2,2), 201	2 (2,2), 178	2.0 (2.0, 3.0), 23	*0.06* ^a^
**PI score**, median (IQR), *n*	6 (5,7), 201	6 (5,7), 178	6.0 (5.0, 9.0), 23	0.38^a^
**Seizures**, *n* (%)	62 (30.8%)	52 (29.2%)	10 (43.5%)	0.16^b^
**Abnormal MRI**, *n* (%)	66 (33.0%)	56 (31.6%)	10 (43.5%)	0.26^b^
**Neonatal infection**, *n* (%)	39 (19.2%)	36 (20.0%)	3 (13.0%)	0.43^b^
**Death**, *n* (%)	10 (5.0%)	6 (3.4%)	4 (17.4%)	***<0.01*** ^b^

*AF, amnionitic fluid; BE, base excess; BW, birth weight; C/S, cesarean section; Chorio, chorioamnionitis; GA, gestational age; IQR, interquartile range; Hct, hematocrit; IUGR, in utero growth restriction; MRI, magnetic resonance imaging; NRBCs, nucleated red blood cells; NRFHT, non-reassuring fetal heart tracing, PI score, Perinatal Insult score*.

a *Wilcoxon rank-sum*.

b *Pearson χ^2^*.

*Bold represent p-value < 0.05*.

### Laboratory Data

Results of the blood gas with lactate (mmol/L) within 1 h of life were adjusted to core body temperature. Lowest (worse) pH and BE measures were used for analysis, as that better represents clinical practice. Biochemistry and hematology data are reported in Table 1. Clinical seizures were confirmed during the first 72 h of life using continuous full-montage video electroencephalogram with interpretation by pediatric neurology. Infection was assigned by clinical or histopathological chorioamnionitis or by proven neonatal sepsis confirmed by positive blood, cerebrospinal fluid, or urine culture with need for >72 h of antibiotics as a surrogate of clinically significant infection.

### Perinatal Insult Score

The Perinatal Insult (PI) score was created by assigning a hierarchical value to the NICHD criteria used to decide initiation of TH to treat HIE. The PI score ranges from 0 to 9 and is calculated by assigning points for severity of metabolic acidosis, need for emergency delivery, 10-min Apgar score <5, assisted ventilation at 10 min of life, and Sarnat scores as previously described (32, 33). This score is linked with greater regional injury on brain MRI (32). For our study, the PI score was used as a global assessment of severity of perinatal HI insult based on the NICHD criteria for TH initiation and to determine its relationship with serum biomarkers.

### Biomarker Measurements

The set of serum biomarkers reported here was chosen from those linked to brain injury in adults and enough supporting evidence in the pediatric population as specified in the Request for Applications (RFA) from the National Institutes of Health, funding the study. Of 201 patients included in the study, 178 had serum clinical remnants collected at admission (prior to TH initiation) and daily from DOL 0 to 6. All serum samples were aliquoted and stored at −80°C. Each aliquot was exposed to only 0–1 thaw/freeze cycle prior to assaying. A custom multiplex enzyme-linked immunosorbent assay (ELISA) was used to measure BDNF, VEGF, IL-6, IL-8, and IL-10 in 5 μl of serum or GFAP in 12 μl of serum in 96-well plate formats [Meso Scale Discovery (MSD), Rockville, MD] as previously described (34). The interassay coefficient of variation for BDNF, VEGF, IL-6, IL-8, IL-10, and GFAP assays were 3.4, 5.9, 5.1, 6.7, 5.6, and 10.9%, respectively. Tau was measured in 5 μl of serum using a commercial ELISA (N451LAA-1, MSD) with an interassay coefficient of variation of 9.0%.

### Statistical Analysis

Data were not normally distributed; thus, results are summarized as median and interquartile range (IQR) for continuous variables. Non-parametric statistical methods were applied to analyze continuous variables including Mann–Whitney *U* or Kruskal–Wallis (KW) with Dunn *post-hoc* tests and Spearman ρ correlations. χ^2^ was applied for categorical variables. Longitudinal biomarker trajectories were analyzed using linear trend of medians (35). Mixed-effect model adjusted for sex to assess the relationships between single indicators of HIE severity (independent variable) and serum biomarkers evaluated for repeated measurements and stratified by time relative to exposure to TH was applied. Additional adjusting for sex and neonatal infection was also modeled. *p* ≤ 0.05 was considered significant. Statistical analysis was performed using GraphPad Prism [version 8.0.0 (131) 2018; GraphPad Software, San Diego, CA] and Stata (version 15, StataCorp LLC, College Station, TX).

## Results

### Demographic, Clinical, and Biochemical Variables

Of 246 infants diagnosed with NE and treated with TH during the study period, 45 met the exclusion criteria as detailed in Figure 1, resulting in 201 patients diagnosed with HIE. Before exclusions, 16 deaths occurred in our cohort (6.5%; 16/246), with 2 deaths not linked to HIE and 4 meeting other exclusion criteria. As a result, the mortality rate for our cohort after exclusions was 5% (10/201, Table 1). Of the 201 infants with HIE treated with TH included in the analysis, 23 did not have serial serum samples for biomarker analysis (Figure 1). Patients without available biomarker data (*n* = 23) had a higher incidence of meconium-stained amniotic fluid (*p* = 0.02), lower Apgar score at 1 min of life (*p* = 0.03), and higher death rate (*p* < 0.01) than patients with biomarkers available (Table 1).

### Longitudinal Changes in Serum Biomarkers

The median (IQR) number of serum samples per patient for biomarker analysis was 5 (3, 7). This resulted in 848 samples analyzed from 178 patients (Table 2). Serum BDNF levels decreased after DOL 0 (first 24 h of life) and recovered to levels at and above admission by DOL 5 (*p* = 0.02, Table 2). Vascular endothelial growth factor levels tended to be lower at admission (before initiation of TH) and recover after 24 h (*p* = 0.19, Table 2). Both tau and GFAP levels tended to peak by DOL 5–6 (*p* = 0.19 and *p* = 0.06, respectively; Table 2). Cytokine levels peaked within the first 24 h of life and decreased thereafter (*p* < 0.001 in all cases, Table 2).

**Table 2:** Longitudinal biomarker levels.

	**DOL**	**Median (IQR)**	***N***			**DOL**	**Median (IQR)**	***N***	
**BDNF** (pg/ml)	0 (Admission)	1,365.3 (630.9, 2,318.5)	85	***p*** = **0.02**	**IL-6** (pg/ml)	0 (Admission)	16.4 (7.54, 54.5)	85	***p*** ***<*** **0.001**
	0	1,308.2 (799.2, 1,883.1)	125			0	21.8 (11.3, 49.5)	126	
	1	1,027.8 (600.4, 1,499.5)	131			1	16.0 (10.1, 30.1)	131	
	2	798.2 (513.6, 1,431.9)	124			2	9.15 (5.63, 16.5)	125	
	3	924.8 (546.2, 1,509.4)	124			3	5.09 (3.23, 10.3)	124	
	4	1,018.5 (662.9, 1,657.6)	85			4	3.35 (1.68, 5.58)	86	
	5	1,360.7 (873.3, 2,177.5)	78			5	2.96 (1.61, 6.28)	79	
	6	1,529.7 (803.6, 2,808.2)	66			6	3.05 (1.59, 8.60)	66	
**VEGF** (pg/ml)	0 (Admission)	36.2 (0.164, 165.4)	83	*p* = 0.19	**IL-8** (pg/ml)	0 (Admission)	83.1 (37.7, 248.8)	80	***p*** ***<*** **0.001**
	0	114.6 (36.1, 214.7)	123			0	102.9 (45, 211.4)	109	
	1	130.5 (71.9, 254.9)	130			1	72.9 (47.8, 155.3)	114	
	2	135.9 (78.2, 232.9)	124			2	60.3 (39.9, 96.5)	104	
	3	141.6 (79.6, 229.7)	123			3	53.2 (35.1, 85.1)	107	
	4	151.5 (85.7, 254.8)	86			4	55.3 (33.6, 94.1)	76	
	5	138.0 (77.5, 232.0)	79			5	45.7 (32.4, 103)	70	
	6	133.7 (80.4, 234.3)	66			6	51.5 (29.6, 126.1)	60	
**Tau** (pg/ml)	0 (Admission)	185.3 (79.9, 386.8)	85	*p* = 0.19	**IL-10** (pg/ml)	0 (Admission)	8.30 (2.67, 43.18)	80	***p*** ***<*** **0.001**
	0	193.5 (84.3, 429.5)	126			0	2.72 (1.16, 8.38)	109	
	1	179.9 (62.7, 452.8)	128			1	1.28 (0.56, 2.96)	114	
	2	178.0 (81.4, 536.6)	121			2	0.763 (0.29, 1.27)	104	
	3	236.7 (112.3, 566.8)	128			3	0.456 (0.06, 0.90)	107	
	4	267.9 (123.2, 598.8)	86			4	0.260 (0.06, 0.70)	76	
	5	300.7 (147.6, 790.2)	74			5	0.312 (0.06, 0.97)	70	
	6	206.5 (95.9, 482.7)	65			6	0.518 (0.06, 1.05)	60	
**GFAP** (ng/ml)	0 (Admission)	0.203 (0.082, 1.079)	89	*p* = 0.06	(IL-6*IL-8) /IL-10 Index	0 (Admission)	177.4 (64.3, 602.7)	80	*p* = 0.99
	0	0.302 (0.071, 1.33)	130			0	984.9 (354.4, 2,459.3)	109	
	1	0.287 (0.072, 1.46)	134			1	1,078.9 (437.9, 5,578.7)	114	
	2	0.353 (0.092, 1.61)	130			2	872.0 (308.3, 4,264.2)	104	
	3	0.371 (0.105, 1.55)	133			3	1,183.7 (322.1, 3,437)	107	
	4	0.581 (0.111, 2.00)	91			4	860.8 (288.7, 3,028.3)	76	
	5	0.769 (0.112, 2.58)	78			5	771.1 (190.9, 3,258.4)	70	
	6	0.712 (0.071, 2.20)	66			6	771 (157.7, 2,392.6)	60	

*Analysis by linear trend of medians for the overall trajectory. Machado and Santos Silva (35). BDNF, brain-derived neurotrophic factor; DOL, day of life; GFAP, glial fibrillary acidic protein; IQR, interquartile range; IL, interleukin; VEGF, vascular endothelial growth factor*.

*Bold represent p-values < 0.05*.

### Correlation Between Clinical Indicators of HIE Severity and Serum Biomarkers

At admission to the NICU, lower 5-min Apgar scores and BE and higher lactate and nucleated red blood cells (NRBCs), all indicators of worse HI insult, correlated with higher Sarnat scores (Table 3). Among the early non-brain-specific indicators of HI insult, higher NRBCs (a marker of prolonged partial *in utero* hypoxia) correlated with lower BDNF and VEGF and higher tau, and cytokines, but not GFAP, whereas higher lactate (a sub-acute HI marker) mostly correlated with lower VEGF and higher tau, GFAP, and cytokines, but not BDNF, during the first week of life (Tables 4A,B). All other early non-brain-specific indicators of HI insult severity (5-min Apgar score, worse pH, and worse BE) mostly correlated with tau (throughout the first week of life) and IL-10 (during the first 72 h of life), explaining the correlation between tau and IL-10 with the PI score, a global assessment of perinatal insult (Tables 4A,B). Higher Sarnat score (worse encephalopathy) correlated with lower VEGF during the first 24 h of life (DOL 0) and higher tau, GFAP, and IL-10 throughout the first week of life (Tables 4A,B). The integration of the proinflammatory (IL-6 and IL-8) and the anti-inflammatory (IL-10) responses in a patient-by-patient basis was suggested by the lost correlation between the (IL-6 ^*^ IL-8)/IL-10 index and most clinical and biochemical indicators of systemic HI severity (i.e., BE, NRBCs, and lactate). Conversely, the persistent correlation between Sarnat scores and this index suggested a disproportionate IL-10 response with worse encephalopathy (Table 4B).

**Table 3:** Correlation between indicators of HI severity [*n* = 178 (subset with biomarker data)].

	**ρ**	***p*-value**	***n***	**ρ**	***p*-value**	***n***	**ρ**	***p*-value**	***n***	**ρ**	***p*-value**	***n***	**ρ**	***p*-value**	***n***
**Apgar 5 min**	**Apgar 5 min**														
	**(*****n*** **=** **172)**														
**Worse pH**	−0.018	0.810	172	•**Worse pH**											
				**(*****n*** **=** **178)**											
**Worse BE**	**0.161**	**0.036**	**171**	**0.527**	**<0.001**	**177**	**Worse BE**								
							•**(*****n*** **=** **177)**								
**NRBCs**	**−0.129**	**0.093**	**170**	−0.113	0.134	176	**−0.215**	**0.004**	**175**	•**NRBCs**					
										•**(*****n*** **=** **176)**					
**Lactate**	**−0.385**	**<0.001**	**104**	**−0.225**	**0.019**	**109**	**−0.374**	**<0.001**	**108**	**0.444**	**<0.001**	**108**	•**Lactate**		
													•**(*****n*** **=** **109)**		
**Sarnat score**	**−0.440**	**<0.001**	**172**	−0.098	0.195	178	**−0.124**	**0.100**	**177**	**0.147**	**0.051**	**176**	**0.326**	**0.001**	**109**

*Analysis by Spearman ρ correlations. BE, base excess; NRBCs, nucleated red blood cells*.

*Bold represent p-values < 0.10*.

**Table 4A:** Correlation between indicators of HI severity and BDNF, VEGF, tau, and GFAP during the first week of life.

		**Apgar 5 min**	**Worse pH**	**Worse BE**	**NRBCs**	**Lactate**	**Sarnat score**	**PI score**
		**(*n* = 172)**	**(*n* = 178)**	**(*n* = 177)**	**(*n* = 176)**	**(*n* = 109)**	**(*n* = 178)**	**(*n* = 178)**
**DOL**		***ρ***	***ρ***	***ρ***	***ρ***	**Rho**	***ρ***	***ρ***
		**(** ***p*** **-value)**	**(** ***p*** **-value)**	**(** ***p*** **-value)**	**(** ***p*** **-value)**	**(** ***p*** **-value)**	**(** ***p*** **-value)**	**(** ***p*** **-value)**
**BDNF**	**0 (A)**	−0.001 (0.99)	−0.04 (0.72)	0.02 (0.85)	***−0.23 (0.03)***	−0.11 (0.41)	0.02 (0.87)	0.04 (0.72)
	**0**	0.10 (0.29)	**0.20 (0.03)**	**0.15 (0.10)**	−0.14 (0.14)	−0.14 (0.22)	−0.07 (0.44)	**−0.16 (0.08)**
	**1**	0.01 (0.95)	**0.17 (0.06)**	0.12 (0.16)	**−0.28 (<0.01)**	−0.08 (0.47)	−0.07 (0.44)	−0.11 (0.23)
	**2**	−0.07 (0.45)	0.09 (0.35)	−0.06 (0.55)	−0.05 (0.59)	0.12 (0.28)	−0.003 (0.97)	0.06 (0.51)
	**3**	−0.07 (0.46)	0.11 (0.24)	0.03 (0.78)	**−0.18 (0.04)**	−0.14 (0.21)	−0.02 (0.87)	−0.09 (0.30)
	**4**	−0.08 (0.47)	**0.23 (0.04)**	0.11 (0.33)	**−0.35 (<0.01)**	−0.13 (0.35)	−0.01 (0.92)	−0.11 (0.34)
	**5**	−0.08 (0.48)	−0.05 (0.66)	0.08 (0.50)	**−0.40 (<0.01)**	−0.15 (0.30)	0.15 (0.21)	0.04 (0.70)
	**6**	0.001 (0.99)	0.03 (0.79)	−0.11 (0.40)	**−0.29 (0.02)**	**−0.28 (0.08)**	0.02 (0.86)	0.01 (0.96)
**VEGF**	**0 (A)**	0.08 (0.50)	0.15 (0.17)	**0.18 (0.10)**	**−0.24 (0.03)**	−0.19 (0.13)	**−0.19 (0.08)**	**−0.19 (0.09)**
	**0**	0.01 (0.89)	**0.21 (0.02)**	**0.34 (<0.01)**	**−0.23 (0.01)**	**−0.33 (<0.01)**	**−0.16 (0.08)**	**−0.17 (0.06)**
	**1**	**0.20 (0.03)**	0.11 (0.22)	**0.29 (<0.01)**	**−0.36 (<0.01)**	**−0.38 (<0.01)**	**−0.19 (0.04)**	−0.12 (0.18)
	**2**	−0.06 (0.52)	−0.06 (0.50)	**0.15 (0.09)**	**−0.27 (<0.01)**	−0.14 (0.21)	−0.06 (0.54)	0.01 (0.94)
	**3**	−0.07 (0.45)	0.13 (0.17)	**0.19 (0.03)**	**−0.35 (<0.01)**	**−0.40 (<0.01)**	0.03 (0.78)	−0.06 (0.53)
	**4**	0.13 (0.26)	0.16 (0.14)	**0.18 (0.09)**	**−0.48 (<0.01)**	**−0.45 (<0.01)**	**−0.23 (0.04)**	−0.13 (0.22)
	**5**	−0.14 (0.22)	0.06 (0.60)	**0.29 (0.01)**	**−0.45 (<0.01)**	**−0.28 (0.04)**	−0.04 (0.75)	−0.03 (0.80)
	**6**	0.09 (0.48)	−0.10 (0.41)	−0.03 (0.81)	**−0.32 (<0.01)**	**−0.32 (0.04)**	−0.09 (0.46)	−0.01 (0.94)
**Tau**	**0 (A)**	−0.10 (0.40)	**−0.19 (0.08)**	−0.16 (0.14)	**0.22 (0.04)**	**0.27 (0.03)**	0.04 (0.74)	0.02 (0.86)
	**0**	**−0.18 (0.05)**	−0.001 (0.99)	−0.06 (0.54)	**0.21 (0.02)**	**0.43 (<0.01)**	**0.19 (0.04)**	0.03 (0.76)
	**1**	**−0.27 (<0.01)**	**−0.20 (0.03)**	**−0.24 (<0.01)**	**0.35 (<0.01)**	**0.41 (<0.01)**	**0.27 (<0.01)**	**0.26 (<0.01)**
	**2**	**−0.24 (<0.01)**	−0.10 (0.29)	**−0.20 (0.03)**	**0.23 (0.01)**	**0.22 (0.05)**	**0.35 (<0.01)**	0.14 (0.14)
	**3**	**−0.22 (0.02)**	**−0.22 (0.01)**	**−0.32 (<0.01)**	**0.21 (0.02)**	**0.19 (0.08)**	**0.31 (<0.01)**	**0.21 (0.02)**
	**4**	0.07 (0.52)	**−0.24 (0.02)**	**−0.19 (0.09)**	0.09 (0.40)	**0.29 (0.03)**	**0.23 (0.04)**	**0.18 (0.10)**
	**5**	−0.12 (0.34)	−0.04 (0.76)	−0.15 (0.19)	0.16 (0.18)	**0.28 (0.04)**	**0.36 (<0.01)**	0.14 (0.24)
	**6**	**−0.31 (0.01)**	**−0.21 (0.09)**	**−0.23 (0.07)**	−0.10 (0.46)	0.15 (0.34)	**0.38 (<0.01)**	**0.27 (0.03)**
**GFAP**	**0 (A)**	**−0.36 (<0.01)**	0.09 (0.40)	−0.001(0.99)	0.10 (0.37)	**0.33 (<0.01)**	**0.20 (0.06)**	0.02 (0.88)
	**0**	−0.10 (0.29)	−0.02 (0.84)	−0.12 (0.18)	0.06 (0.47)	**0.28 (0.01)**	0.11 (0.23)	0.06 (0.49)
	**1**	**−0.30 (<0.01)**	−0.11 (0.21)	**−0.16 (0.07)**	0.10 (0.27)	**0.34 (<0.01)**	**0.22 (0.01)**	**0.18 (0.04)**
	**2**	**−0.22 (0.02)**	−0.05 (0.57)	−0.09 (0.31)	0.06 (0.52)	**0.21 (0.06)**	**0.22 (0.01)**	0.10 (0.28)
	**3**	−0.14 (0.12)	−0.13 (0.14)	**−0.14 (<0.10)**	0.09 (0.30)	0.16 (0.13)	**0.21 (0.02)**	**0.16 (0.07)**
	**4**	0.07 (0.55)	**−0.18 (0.09)**	**−0.21 (0.05)**	0.02 (0.88)	**0.28 (0.03)**	0.16 (0.13)	0.15 (0.17)
	**5**	−0.12 (0.34)	−0.13 (0.25)	−0.12 (0.28)	0.004 (0.98)	**0.36 (<0.01)**	**0.38 (<0.01)**	**0.24 (0.04)**
	**6**	**−0.27 (0.04)**	−0.08 (0.52)	−0.18 (0.15)	−0.20 (0.11)	**0.26 (0.10)**	**0.21 (0.10)**	0.14 (0.27)

*Analysis by Spearman ρ correlation. A, admission; BDNF, brain-derived neurotrophic factor; BE, base excess; DOL, day of life; GFAP, glial fibrillary acidic protein; NRBCs, nucleated red blood cells; PI score, Perinatal Insult score; VEGF, vascular endothelial growth factor*.

*Bold represent p-values < 0.10*.

**Table 4B:** Correlation between indicators of HI severity and cytokines during the first week of life.

		**Apgar 5 min**	**Worse pH**	**Worse BE**	**NRBCs**	**Lactate**	**Sarnat score**	**PI score**
		**(*n* = 172)**	**(*n* = 178)**	**(*n* = 177)**	**(*n* = 176)**	**(*n* = 109)**	**(*n* = 178)**	**(*n* = 178)**
**DOL**		***ρ***	***ρ***	***ρ***	***ρ***	**Rho**	***ρ***	***ρ***
		**(** ***p*** **-value)**	**(** ***p*** **-value)**	**(** ***p*** **-value)**	**(** ***p*** **-value)**	**(** ***p*** **-value)**	**(** ***p*** **-value)**	**(** ***p*** **-value)**
**IL-6**	**0 (A)**	**−0.19 (0.09)**	−0.11 (0.31)	−0.18 (0.11)	**0.40 (<0.01)**	**0.25 (0.05)**	**0.21 (0.06)**	0.17 (0.13)
	**0**	−0.15 (0.11)	−0.11 (0.24)	−0.09 (0.35)	**0.17 (0.06)**	0.07 (0.53)	0.06 (0.53)	0.04 (0.69)
	**1**	0.10 (0.28)	**−0.18 (0.05)**	**−0.19 (0.03)**	0.06 (0.54)	0.08 (0.46)	−0.01 (0.95)	0.09 (0.34)
	**2**	0.01 (0.88)	−0.15 (0.10)	**−0.16 (0.08)**	**0.28 (<0.01)**	**0.19 (0.09)**	0.03 (0.76)	0.03 (0.74)
	**3**	0.001 (0.99)	**−0.25 (<0.01)**	−0.12 (0.19)	**0.18 (0.05)**	0.10 (0.37)	0.07 (0.47)	0.10 (0.27)
	**4**	−0.02 (0.86)	−0.15 (0.17)	−0.05 (0.63)	**0.32 (<0.01)**	**0.35 (<0.01)**	−0.16 (0.14)	0.09 (0.42)
	**5**	0.01 (0.91)	−0.07 (0.56)	−0.03 (0.80)	**0.40 (<0.01)**	**0.24 (0.08)**	−0.01 (0.95)	−0.01 (0.96)
	**6**	−0.05 (0.73)	−0.16 (0.21)	−0.03 (0.81)	**0.23 (0.07)**	0.26 (0.11)	0.12 (0.36)	0.15 (0.25)
**IL−8**	**0 (A)**	**−0.22 (0.06)**	−0.03 (0.81)	**−0.26 (0.02)**	**0.52 (<0.01)**	**0.34 (<0.01)**	0.15 (0.18)	0.14 (0.21)
	**0**	**−0.23 (0.02)**	−0.07 (0.49)	**−0.18 (0.07)**	**0.38 (<0.01)**	**0.26 (0.03)**	**0.22 (0.03)**	**0.22 (0.02)**
	**1**	0.04 (0.71)	**−0.21 (0.03)**	−0.13 (0.19)	**0.24 (0.01)**	**0.33 (<0.01)**	0.05 (0.62)	0.09 (0.35)
	**2**	−0.11 (0.29)	−0.11 (0.27)	−0.11 (0.29)	**0.26 (<0.01)**	**0.33 (<0.01)**	−0.03 (0.76)	0.01 (0.92)
	**3**	−0.01 (0.92)	−0.05 (0.60)	−0.02 (0.82)	0.04 (0.67)	0.14 (0.22)	−0.05 (0.63)	−0.03 (0.79)
	**4**	−0.08 (0.50)	−0.02 (0.87)	0.09 (0.45)	0.04 (0.72)	0.004 (0.98)	−0.17 (0.14)	−0.09 (0.45)
	**5**	−0.09 (0.47)	−0.07 (0.58)	−0.08 (0.50)	**0.25 (0.04)**	**0.35 (0.01)**	0.04 (0.76)	0.07 (0.59)
	**6**	**−0.30 (0.02)**	−0.03 (0.81)	−0.05 (0.71)	0.11 (0.42)	**0.37 (0.02)**	0.16 (0.23)	0.11 (0.39)
**IL−10**	**0 (A)**	−0.14 (0.23)	−0.07 (0.55)	**−0.22 (0.06)**	**0.51 (<0.01)**	**0.29 (0.02)**	**0.23 (0.04)**	**0.19 (0.10)**
	**0**	**−0.23 (0.02)**	−0.03 (0.78)	**−0.25 (0.01)**	**0.48 (<0.01)**	**0.39 (<0.01)**	**0.25 (0.01)**	**0.20 (0.04)**
	**1**	−0.10 (0.32)	−0.10 (0.29)	**−0.30 (<0.01)**	**0.34 (<0.01)**	**0.25 (0.03)**	**0.19 (0.05)**	**0.21 (0.03)**
	**2**	−0.12 (0.26)	−0.01 (0.95)	**−0.17 (0.10)**	**0.36 (<0.01)**	**0.25 (0.04)**	**0.30 (<0.01)**	**0.17 (0.09)**
	**3**	0.08 (0.41)	−0.11 (0.28)	−0.10 (0.30)	**0.19 (0.05)**	0.14 (0.24)	**0.19 (0.05)**	0.05 (0.59)
	**4**	0.02 (0.88)	−0.03 (0.77)	0.14 (0.24)	**0.21 (0.08)**	0.19 (0.18)	0.17 (0.13)	0.11 (0.36)
	**5**	−0.03 (0.83)	−0.09 (0.45)	−0.13 (0.28)	**0.21 (0.08)**	**0.25 (0.08)**	0.12 (0.32)	0.15 (0.21)
	**6**	−0.11 (0.42)	0.08 (0.56)	0.05 (0.72)	0.16 (0.23)	**0.28 (0.09)**	**0.26 (0.05)**	0.08 (0.53)
(IL−6*IL−8)/IL−10 index	**0 (A)**	−0.17 (0.16)	0.03 (0.78)	−0.02 (0.89)	0.03 (0.81)	0.003 (0.98)	0.05 (0.68)	0.03 (0.83)
	**0**	−0.12 (0.25)	−0.04 (0.71)	0.02 (0.81)	−0.03 (0.75)	−0.12 (0.31)	0.01 (0.95)	−0.01 (0.96)
	**1**	**0.23 (0.02)**	−0.15 (0.11)	0.11 (0.25)	−0.15 (0.12)	−0.02 (0.85)	**−0.19 (0.05)**	−0.11 (0.24)
	**2**	0.08 (0.45)	−0.07 (0.46)	0.05 (0.63)	−0.08 (0.42)	0.02 (0.87)	**−0.26 (<0.01)**	−0.14 (0.15)
	**3**	−0.05 (0.65)	−0.03 (0.75)	0.09 (0.36)	**−0.16 (0.10)**	−0.05 (0.64)	−0.13 (0.18)	−0.01 (0.92)
	**4**	−0.004 (0.97)	−0.08 (0.49)	−0.13 (0.28)	−0.003 (0.98)	0.05 (0.71)	**−0.30 (<0.01)**	−0.05 (0.65)
	**5**	−0.02 (0.86)	0.06 (0.64)	0.14 (0.25)	**0.24 (0.05)**	0.12 (0.40)	−0.11 (0.38)	−0.10 (0.40)
	**6**	−0.004 (0.98)	−0.18 (0.17)	−0.16 (0.24)	0.16 (0.23)	0.22 (0.19)	−0.10 (0.46)	0.09 (0.48)

*Analysis by Spearman ρ-correlation. A, admission; BE, base excess; DOL, day of Life; IL, interleukin; NRBCs, nucleated red blood cells; PI score, perinatal Insult score*.

*Bold represent p-values < 0.10*.

### Stratification of Biomarker Trajectories by Sarnat Score

**Figure 2:**
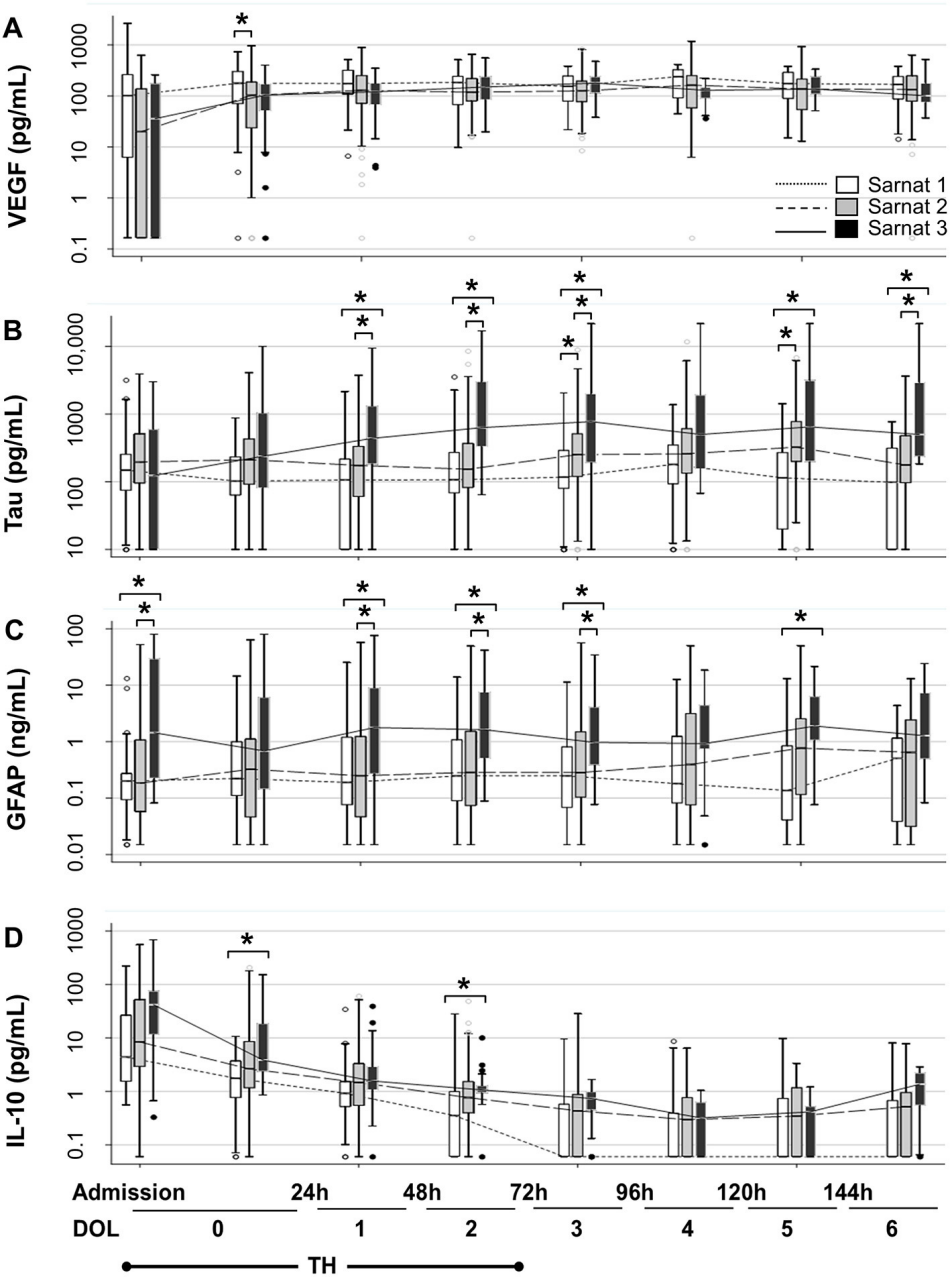
Temporal trajectories of Sarnat score–stratified serum biomarkers. Temporal trajectories of VEGF **(A)**, tau **(B)**, GFAP **(C)**, and IL-10 **(D)** levels during the first 7 days of life (DOL 0–6) stratified by Sarnat score (1, white; 2, gray, and 3, black) are shown. Two specimens were collected during first 24 h of life (DOL 0), the first sample obtained after admission and the one collected in the second 12 h of life. Data are represented as box-and-whiskers plot, where the box represents the IQR limited by the 25th and 75th percentiles, with solid line inside the box representing the median. Whiskers are limited by the last data point sitting within 1.5 times the IQR, whereas outliers are represented as circles beyond the boundaries of the whiskers. The biomarker level in a 10-base log scale is shown in the *y* axis. Time in hours and DOL for all panels are depicted in the bottom of the figure. TH exposure occurred for 72 h upon admission to the NICU (for simplification DOL 0–2). *p < 0.05 (KW analysis of variance with Dunn Bonferroni *post-hoc* analysis). DOL, day of life; GFAP, glial fibrillary acidic protein; IL, interleukin; TH, therapeutic hypothermia; VEGF, vascular endothelial growth factor.

Sarnat scores were inversely correlated with VEGF and directly with tau, GFAP, and IL-10 (Tables 4A,B). Patients with Sarnat scores of 2 and 3 had a VEGF nadir soon after admission, which recovered after 24 h of life [*p* < 0.001 (Sarnat 2), *p* = 0.008 (Sarnat 3); Figure 2A]. Unlike VEGF, GFAP discriminated between patients with Sarnat scores of 2 and 3. Only patients with Sarnat scores of 3 had higher GFAP levels since admission to the NICU (*p* = 0.03 vs. Sarnat 1; *p* = 0.02 vs. Sarnat 2; KW *p* = 0.04) and throughout the first 72 h of life (*p* = 0.003, Figure 2C). Tau, the other marker of neural cell injury, only began to increase after the first 24 h of life (DOL 1) in patients with Sarnat score of 3, reaching levels fourfold to sixfold higher than those in patients with lower scores by 48 to 72 h of life (DOL 2) (*p* < 0.001 vs. either Sarnat 1 or Sarnat 2; Figure 2B). Conversely, patients with score of 2 only began to increase their tau and GFAP levels around the time of TH completion (72 h of life, DOL 2), resulting in approximately three-fold [*p* = 0.01 (tau)] and approximately six-fold [*p* = 0.07 (GFAP)] higher levels than those in patients with scores of 1 [*p* = 0.006 (tau) and 0.004 (GFAP)] by DOL 5 (Figures 2B,C). At admission, patients with Sarnat scores of 3 also tended to have higher IL-10 levels than those with Sarnat scores of 1 (*p* = 0.05; Figure 2D). Although IL-10 levels decreased after the first 24 h of life (*p* < 0.001 for all Sarnat groups), they remained relatively elevated in patients with Sarnat scores of 2 and 3, and by DOL 2 (48–72 h of life) prior to completion of TH, IL-10 levels were more than twice the levels in patients with Sarnat of 1 (*p* = 0.009; Figure 2D).

### Adjusted Relationship Between Severity of HI Insult and Serum Biomarkers

**Figure 3:**
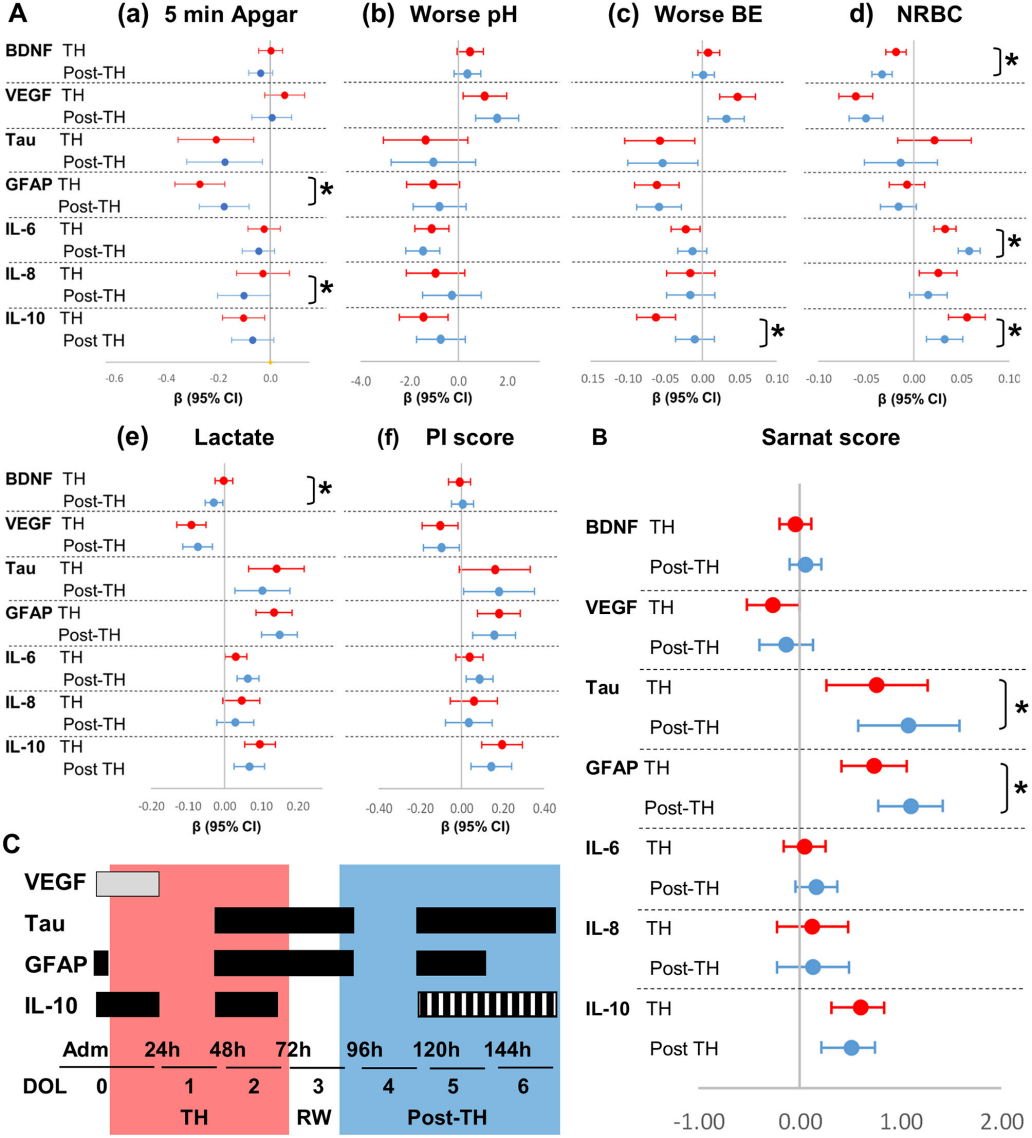
Forest plots for adjusted associations between HI severity indicators and serum biomarkers by exposure to TH. Forest plots represent relationships adjusted for sex between **(A)** indicators of severity of HI insult and **(B)** Sarnat scores with biomarkers levels during (DOL 0–2, red) and after (DOL 4–6, blue) TH. Admission and DOL 3 (TH-rewarming) data were not included in the adjusted analysis intended to assess the effect of TH in trajectories. β-Coefficients reaching *p*-values < 0.05 in mixed model analysis adjusted for sex do not cross line at zero. *p-value comparing during and after TH periods is shown. The strength of associations, as determined by the β coefficients and CI (95%), is assessed by the distance from zero (reference line). **(C)** Proposed timeline to maximize utility of serum biomarkers against Sarnat score. Time intervals of maximum utility are shown in horizontal boxes in gray for levels expected to decrease and in black for levels expected to increase with higher HI brain injury. Red and blue zones represent the period during and after TH, respectively. Hashed box for IL-10 represent speculated increased levels with higher HI injury.

After adjustment for sex [Figures 3A_(a−*f*)_,B; Supplementary Table 1] and infection (Table 5; Supplementary Table 2) and stratification by TH (DOL 0–2) and post-TH (DOL 4–6) periods, most relationships between indicators of HI insult severity and serum biomarkers persisted as described above. Tau and GFAP had the strongest association with 5-min Apgar score [Figure 3A_(a)_], worse BE [Figure 3A_(c)_], and lactate [Figure 3A_(e)_], but lacked association with NRBCs [Figure 3A_(d)_], during and after TH. Lower VEGF during TH and higher tau, GFAP, and IL-10 during and after TH were most strongly associated with worse Sarnat score [Figure 3B]. Some of these relationships changed during and after TH. For example, lower 5-min Apgar scores were associated with higher GFAP, but this association was 75% stronger during TH than after TH [Figure 3A_(a)_], despite higher GFAP levels after TH (Table 2). In addition, the association between lower BE and higher IL-10 levels was six-fold stronger during TH than after TH [Figure 3A_(c)_]. Conversely, the strength of association between lower 5-min Apgar and higher IL-8 [Figure 3A_(a)_] and higher NRBCs and higher IL-6 [Figure 3A_(d)_] became stronger after TH. Lastly, the associations between tau and GFAP with Sarnat scores became 50% stronger after TH [Figure 3B]. Adjustment for infection did not change most relationships between indicators of severity of HI insult and cytokines (Supplementary Table 2).

**Table 5:** Summary of mixed-model adjustments for indicators of HI severity, time of biomarker, sex, and neonatal infection status.

			**β (95% CI)**	***p*-value**	**Inter-*p*-value**
**Apgar 5 min**	**VEGF**	TH	0.052 (−0.029, 0.133)	0.211	0.305
		Post-TH	0.003 (−0.097, 0.103)	0.951	
	**Tau**	TH	−0.211 (−0.324, −0.098)	**<0.001**	0.537
		Post-TH	−0.177 (−0.308, −0.047)	**0.008**	
	**GFAP**	TH	−0.273 (−0.418, −0.128)	**<0.001**	**0.042**
		Post-TH	−0.179 (−0.336, −0.022)	0.025	
	**IL-10**	TH	−0.101 (−0.205, 0.004)	0.06	0.542
		Post-TH	−0.063 (−0.191, 0.064)	0.331	
**Worse pH**	**VEGF**	TH	0.994 (0.003, 1.984)	0.049	0.375
		Post-TH	1.476 (0.284, 2.667)	**0.015**	
	**Tau**	TH	−1.344 (−2.670, −0.019)	0.047	0.624
		Post-TH	−1.036 (−2.541, 0.470)	0.178	
	**GFAP**	TH	−1.041 (−2.774, 0.692)	0.239	0.623
		Post-TH	−0.790 (−2.635, 1.054)	0.401	
	**IL-10**	TH	−1.305 (−2.501, −0.109)	0.032	0.296
		Post-TH	−0.591 (−2.034, 0.852)	0.422	
**Base excess**	**VEGF**	TH	0.045 (0.019, 0.071)	**0.001**	0.288
		Post-TH	0.029 (−0.003, 0.061)	**0.08**	
	**Tau**	TH	−0.058 (−0.093, −0.024)	**0.001**	0.811
		Post-TH	−0.054 (−0.094, −0.015)	**0.007**	
	**GFAP**	TH	−0.062 (−0.110, −0.015)	**0.01**	0.806
		Post-TH	−0.059 (−0.109, −0.009)	**0.022**	
	**IL-10**	TH	−0.058 (−0.093, −0.024)	**0.001**	**0.007**
		Post-TH	−0.006 (−0.047, 0.034)	0.758	
**NRBCs**	**VEGF**	TH	−0.058 (−0.077, −0.038)	**<0.001**	0.305
		Post-TH	−0.048 (−0.068, −0.028)	**<0.001**	
	**Tau**	TH	0.022 (−0.007, 0.052)	0.139	**0.001**
		Post-TH	−0.013 (−0.043, 0.018)	0.406	
	**GFAP**	TH	−0.007 (−0.046, 0.032)	0.738	0.312
		Post-TH	−0.016 (−0.055, 0.024)	0.433	
	**IL-10**	TH	0.052 (0.026, 0.078)	**<0.001**	**0.072**
		Post-TH	0.029 (0.002, 0.056)	**0.033**	
**Lactate**	**VEGF**	TH	−0.084 (−0.125, −0.042)	**<0.001**	0.447
		Post-TH	−0.066 (−0.117, −0.015)	**0.011**	
	**Tau**	TH	0.143 (0.085, 0.200)	**<0.001**	0.138
		Post-TH	0.106 (0.041, 0.170)	**0.001**	
	**GFAP**	TH	0.135 (0.058, 0.211)	**0.001**	0.483
		Post-TH	0.150 (0.069, 0.231)	**<0.001**	
	**IL-10**	TH	0.090 (0.035, 0.144)	**0.001**	0.311
		Post-TH	0.060 (−0.005, 0.124)	0.07	
**Sarnat score**	**VEGF**	TH	−0.246 (−0.536, 0.043)	0.096	0.412
		Post-TH	−0.116 (−0.467, 0.235)	0.517	
	**Tau**	TH	0.773 (0.407, 1.139)	**<0.001**	0.071
		Post-TH	1.088 (0.663, 1.513)	**<0.001**	
	**GFAP**	TH	0.744 (0.244, 1.243)	**0.004**	**0.014**
		Post-TH	1.105 (0.574, 1.637)	**<0.001**	
	**IL-10**	TH	0.577 (0.216, 0.939)	**0.002**	0.657
		Post-TH	0.487 (0.051, 0.922)	0.029	

*Mixed-model analysis with adjustments. GFAP, glial fibrillary acidic protein; NRBCs, nucleated red blood cells; PI score, perinatal Insult score; TH, therapeutic hypothermia; VEGF, vascular endothelial growth factor*.

*Bold represent p-values < 0.05*.

## Discussion

The strength of the relationships between serum biomarkers and clinical indicators of HIE severity is modulated by TH. Here, we show that after adjusting for sex and infection, lower BE and higher lactate (biochemical markers of decreased perfusion), and worse Sarnat scores (functional outcome) all relate to lower VEGF and higher tau, GFAP, and IL-10. Within the first 24 h of life, patients with Sarnat scores of 2–3 have lower VEGF, whereas those with scores of 3 also have high GFAP and IL-10. Thus, measuring VEGF, GFAP, and IL-10 soon after admission to the NICU may assist to stratify more accurately moderate–severe HI brain injury. During the next 72 h (TH period), tau levels begin to increase. However, while in patients with a Sarnat score of 3 the increase in tau begins on DOL 0–1, in those with a score of 2, this increase is delayed until DOL 2–3. By 72 h after completion of TH (DOL 5), patients with Sarnat scores of 2 reach tau, GFAP, and IL-10 levels similar to those of patients with scores of 3. The increase in tau and GFAP levels after TH may reflect the role of TH in delaying activation of injurious pathways, which are the target of multiple adjuvant therapies under study (36–38). In adjusted models, the associations between Sarnat scores with tau and GFAP become stronger after TH. Whether these associations represent persistent or worsening HI brain injury after completion of TH requires further investigation. In light of our data, we propose a screening schedule of HIE patients using VEGF, tau, GFAP, and IL-10 at specific time points related to TH (Figure 3C).

Hypoxic-ischemic encephalopathy severity is traditionally assessed by a combination of clinical and biochemical indicators, which, despite their lack of brain specificity, are used to guide TH initiation. Although Apgar and Sarnat scores are subjective (25, 39), the Sarnat score remains among the most brain-specific indicator available to assess clinically HI injury (40). Other indicators available at admission to the NICU, such as pH, BE, NRBCs, and lactate levels, are systemic markers of decreased fetal perfusion and are not brain-specific. Scores providing a global assessment of severity of perinatal insult, such the PI score (32), lack sensitivity and specificity to HI brain injury; whereas cerebral oximetry and electroencephalographic activity, which are more specific indicators of brain injury, are not broadly used due to the expertise required for their interpretation (41, 42). Thus, circulating biomarkers for HI brain injury may improve the ability of clinical and biochemical indicators alone to assess severity of injury. However, a better understanding of how TH affects their relationships is needed.

Among the molecules measured for this study, tau and GFAP are the most nervous system-specific proteins, which get spilled to the blood stream upon injury of neurons and astrocytes, respectively (34, 43). Because of their different cellular origins, it is not surprising that they have different temporal trajectories. Glial fibrillary acidic protein can discriminate Sarnat 3 from lower scores at admission to the NICU, whereas tau levels peak until the end of TH in neonates with Sarnat 3 and after TH in those with score of 2. Glial fibrillary acidic protein levels did not discriminate between Sarnat 1 and 2 at any time point. This may be due to the sensitivity of the study to detect differences, as only ~20% of the cohort had a Sarnat of 1, or may reflect similar levels of astrocyte injury in these two groups. When adjusted for sex and infection, worse Sarnat score was associated with higher tau and GFAP levels, more so after TH. Whether patients with more severe HI brain insults develop worsening injury with increasing tau and GFAP levels after completion of TH needs further investigation. Our results agree with other studies that have confirmed the direct association between Sarnat scores and circulating tau (19, 22, 23, 34, 44) and GFAP (21, 24, 34). However, our study is the first to address the potential role of TH/rewarming in the temporal trajectory of these biomarkers.

Vascular endothelial growth factor supports the blood–brain barrier (BBB) after neonatal HI brain injury (45), but its production may be compromised with severe insults. The association between high Sarnat score and decreased VEGF levels during TH shown here may suggest that worse HI brain insults may impair VEGF production. Associations between VEGF and early non-brain-specific indicators, such as low pH and BE and high lactate and NRBCs, persist before and after TH. One possible explanation is that injury of other organ systems may carry a significant influence in circulating VEGF levels, particularly after TH. As a result, the early decrease in VEGF may be primarily cause by HI brain injury with some systemic influence, whereas any deficit seen after TH may be mostly linked to persistent systemic compromise. The influence of the recovery of the BBB microstructure in these relationships after TH (46) remains unexplored. In preclinical models, neonatal HI injury leads to delayed regional decrease in BDNF levels (47, 48), the second neurotrophin studied here. In agreement, we report a decrease in serum BDNF levels in infants after HIE, which shows nonetheless no association with Sarnat scores. In a smaller cohort, Massaro et al. reported correlation between low BDNF levels and worse neurodevelopmental outcomes at age 1, but the analysis was unadjusted, limiting interpretation (19). In a larger cohort, higher Sarnat scores were linked to lower BDNF levels (34), an association likely powered by the inclusion of a group with a Sarnat score of 0. As infants with Sarnat scores of 0 are rarely assessed for TH, from a translation perspective, our results suggest that VEGF is a better candidate than BDNF for predictive modeling in the NICU. Our finding that high NRBCs relate to low BDNF may suggest a link with partial prolonged hypoxic events *in utero*.

Cytokines, such as IL-6 (15, 29, 49), IL-8 (21, 49), and IL-10 (49, 50), were the first peripheral blood and cerebrospinal fluid biomarkers described for HIE (51), but validation against multiple indicators of severity of HI in a longitudinal infection-adjusted model relative to TH has not been done in a cohort this large. There is a strong association between Sarnat scores with IL-10 during and after TH; however, such associations do not exist with IL-6 and IL-8. Circulating IL-10 may be the best initial biomarker of severity of HI insult to the brain among the chosen cytokines. Conversely, the infection-adjusted associations of non-specific indicators of severe HI insult with proinflammatory cytokines, IL-6 and IL-8, may suggest a systemic inflammatory response, which becomes stronger after TH is completed. Thus, previous associations between higher IL-6 and IL-8 with worse neurodevelopmental outcomes in HIE patients (52) may not be directly linked to severity of brain injury but instead to the degree of multiorgan involvement leading to systemic inflammation, which influences final brain outcomes (51). Altogether, worse global HI injury, not necessarily to the brain, may lead to an early inflammatory response with cytokine release, including IL-10, a response that may persist or worsen after TH because of injury to other organs. Higher IL-6, IL-8, and IL-10 on DOL 0 may help determine severity of global HI insult, whereas persistent elevation of IL-10 before completion of TH and thereafter, in combination with tau and GFAP, may guide the assessment of ongoing brain injury.

Our study has several limitations. Although recent reports have not identified associations between timing of TH initiation and dysfunctional cerebral autoregulation (32), injury on brain MRI or neurodevelopmental outcomes (53), delay in the initiation of TH may influence the trajectory of the proposed biomarkers. In addition, systemic hypotension and disturbed autoregulation, hypoxia, and hypercapnia may further increase brain injury after HI and influence the strength of associations between Sarnat scores with tau and GFAP. Clinical practice drift has occurred over the course of this study (2009–2019), but the influence of these changes on the longitudinal trajectory of serum biomarker presented here is difficult to isolate. We also were limited by blood volume available for analysis preventing quantification of other biomarkers previously studied in association with neonatal HIE. Although this is one of the largest biomarker studies in neonatal HIE and ~90% of the whole cohort had serum samples available for analysis, selection bias is still likely, as a subset of the sicker patients lacked longitudinal blood samples to measure biomarkers. While freezing and thawing of samples were limited, the samples used in the study were frozen for several years, which may have led to sample degradation, which could impact biomarker levels. Lastly, the usefulness of the proposed biomarkers in predicting the outcomes of patients treated with TH for HIE needs future studies.

## Conclusions

Understanding the influence of TH on the temporal trajectory of serum biomarkers commonly tested to stratify severity of neonatal HIE is an essential step in developing precision in diagnosis of brain injury and building of predictive models. We show the temporal effect of TH in the relationships between serum biomarkers and traditional indicators of severity of HI insult to the brain and other organs. Admission and the period before completion of TH and rewarming may prove to be optimal time points to use biomarkers to stratify patients to modified and novel adjuvant therapeutic strategies. Considering our analysis, we proposed screening of HIE patients at three critical time points: (i) within the first 24 h of life using VEGF, GFAP, and IL-10; (ii) before completion of TH using tau, GFAP, and IL-10; and (iii) by 72 h after completion of TH with tau and GFAP (Figure 3C). These time-sensitive serum biomarkers would improve the stratification of severity of HI brain injury to assign patients to novel neuroprotective or restorative therapies for HIE and to follow therapeutic response.

## Data Availability Statement

The original contributions presented in the study are included in the article/Supplementary Material, further inquiries can be directed to the corresponding author/s.

## Ethics Statement

The studies involving human participants were reviewed and approved by Johns Hopkins School of Medicine IRB. Written informed consent to participate in this study was provided by the participants' legal guardian/next of kin.

## Author Contributions

RC-V, DV, CP, GG, AT, SB, EG, FN, and AE: conceptualization and design of the study. RC-V, SM, HS, DV, FN, and AE: methodology. RC-V, CP, BD, EG, FN, and AE: supervision and oversight. RC-V, FN, and AE: funding acquisition. RC-V and DV: formal data analysis. RC-V, DV, CP, BD, EG, GG, AT, SB, FN, and AE: resources. RC-V, SM, HS, and AE: drafting of significant portions of manuscript, tables, and figures. All authors: data acquisition, review, and editing of final manuscript.

## Funding

This work was supported by National Institutes of Health RO1HD086058 (AE, FN, EG, DV, CP, AT, GG, and SB); RO1HD070996, AG061643, NS109029, and HD074593-07 (FN); KO8NS096115 and 3K08NS096115-03S1 (RC-V); the JHU-SOM Clinician Scientist Award (RC-V); the Thomas Wilson Foundation (RC-V). Under a license agreement between ImmunArray Ltd. and the Johns Hopkins University, the University and AE are entitled to royalties on an invention described in this study and discussed in this publication.

## Conflict of Interest

The authors declare that the research was conducted in the absence of any commercial or financial relationships that could be construed as a potential conflict of interest.

## Publisher's Note

All claims expressed in this article are solely those of the authors and do not necessarily represent those of their affiliated organizations, or those of the publisher, the editors and the reviewers. Any product that may be evaluated in this article, or claim that may be made by its manufacturer, is not guaranteed or endorsed by the publisher.
