# Supplementary material for: Therapeutic Hypothermia Modulates the Relationships Between Indicators of Severity of Neonatal Hypoxic Ischemic Encephalopathy and Serum Biomarkers
Source: Front Neurol. 2021 Nov 2;12:748150. doi: 10.3389/fneur.2021.748150 (PMC8593186; doi:10.3389/fneur.2021.748150)
Supplement: Supplementary file 1 [file Data_Sheet_1.docx]

**Supplemental Material**

1. Table IVA. Mixed model adjusted for indicators of HI severity, time of biomarker (repeated measures) and sex
2. Table IVB. Mixed model adjusted for indicators of HI severity, time of biomarker (repeated measures), sex, and neonatal infection status

**Table S1. Mixed model adjusted for indicators of HI severity, time of biomarker**

**(repeated measures) and sex**

| **Apgar at 5 min** | |  |  |  |  |
| --- | --- | --- | --- | --- | --- |
| **TH Status** | **Biomarker** | **β Coefficient ± SE** | **p-value** | **95% CI** | **Inter-p-value** |
| TH | BDNF | 0.002 ± 0.024 | 0.926 | (-0.044, 0.048) | 0.235 |
| Post-TH |  | -0.037 ± 0.031 | 0.238 | (-0.099, 0.025) |  |
| TH | VEGF | 0.055 ± 0.042 | 0.185 | (-0.026, 0.137) | 0.300 |
| Post-TH |  | 0.006 ± 0.051 | 0.906 | (-0.095, 0.107) |  |
| TH | TAU | ***-0.208 ± 0.058*** | ***<0.001*** | ***(-0.321, -0.095)*** | 0.543 |
| Post-TH |  | ***-0.175 ± 0.067*** | ***0.009*** | ***(-0.305, -0.045)*** |  |
| TH | GFAP | ***-0.271 ± 0.074*** | ***<0.001*** | ***(-0.417, -0.126)*** | ***0.041*** |
| Post-TH |  | ***-0.177 ± 0.080*** | ***0.026*** | ***(-0.334, -0.021)*** |  |
| TH | IL6 | -0.024 ± 0.039 | 0.541 | (-0.101, 0.053) | 0.642 |
| Post-TH |  | -0.045 ± 0.049 | 0.359 | (-0.140, 0.051) |  |
| TH | IL8 | -0.028 ± 0.033 | 0.401 | (-0.092, 0.037) | ***0.048*** |
| Post-TH |  | ***-0.101 ± 0.040*** | ***0.012*** | ***(-0.180, -0.023)*** |  |
| TH | IL10 | -0.103 ± 0.054 | 0.055 | (-0.209, 0.002) | 0.556 |
| Post-TH |  | -0.068 ± 0.066 | 0.302 | (-0.196, 0.061) |  |
| TH | (IL6*IL8)/IL10 Index | 0.081 ± 0.061 | 0.186 | (-0.039, 0.200) | 0.152 |
| Post-TH |  | -0.028 ± 0.076 | 0.716 | (-0.178, 0.122) |  |
|  |  |  |  |  |  |
| **pH** |  |  |  |  |  |
| **TH Status** | **Biomarker** | **β Coefficient ± SE** | **p-value** | **95% CI** | **Inter-p-value** |
| TH | BDNF | 0.483 ± 0.275 | 0.079 | (-0.056, 1.022) | 0.778 |
| Post-TH |  | 0.376 ± 0.356 | 0.291 | (-0.322, 1.074) |  |
| TH | VEGF | ***1.088 ± 0.510*** | ***0.033*** | ***(0.088, 2.088)*** | 0.353 |
| Post-TH |  | ***1.593 ± 0.612*** | ***0.009*** | ***(0.394, 2.792)*** |  |
| TH | TAU | ***-1.329 ± 0.675*** | ***0.049*** | ***(-2.652, -0.007)*** | 0.618 |
| Post-TH |  | -1.016 ± 0.766 | 0.185 | (-2.518, 0.485) |  |
| TH | GFAP | -1.019 ± 0.882 | 0.248 | (-2.747, 0.709) | 0.622 |
| Post-TH |  | -0.768 ± 0.938 | 0.413 | (-2.608, 1.071) |  |
| TH | IL6 | ***-1.084 ± 0.452*** | ***0.017*** | ***(-1.970, -0.197)*** | 0.481 |
| Post-TH |  | ***-1.445 ± 0.552*** | ***0.009*** | ***(-2.528, -0.363)*** |  |
| TH | IL8 | ***-0.933 ± 0.365*** | ***0.011*** | ***(-1.648, -0.218)*** | 0.106 |
| Post-TH |  | -0.263 ± 0.442 | 0.551 | (-1.129, 0.603) |  |
| TH | IL10 | ***-1.415 ± 0.614*** | ***0.021*** | ***(-2.618, -0.212)*** | 0.304 |
| Post-TH |  | -0.712 ± 0.739 | 0.335 | (-2.161, 0.737) |  |
| TH | (IL6*IL8)/IL10 Index | -0.540 ± 0.686 | 0.431 | (-1.884, 0.803) | 0.655 |
| Post-TH |  | -0.928 ± 0.857 | 0.279 | (-2.608, 0.752) |  |

| **Table S1. (Continuation).** **Mixed model adjusted for indicators of HI severity, time of biomarker (repeated measures) and sex** | | | | | |
| --- | --- | --- | --- | --- | --- |
| **Base Excess** | |  |  |  |  |
| **TH Status** | **Biomarker** | **β Coefficient ± SE** | **p-value** | **95% CI** | **Inter-p-value** |
| TH | BDNF | 0.008 ± 0.007 | 0.255 | (-0.006, 0.023) | 0.455 |
| Post-TH |  | 0.001 ± 0.010 | 0.949 | (-0.019, 0.020) |  |
| TH | VEGF | ***0.047 ± 0.013*** | ***<0.001*** | ***(0.021, 0.073)*** | 0.316 |
| Post-TH |  | 0.032 ± 0.017 | 0.052 | (<0.001, 0.065) |  |
| TH | TAU | ***-0.057 ± 0.018*** | ***0.001*** | ***(-0.091, -0.022)*** | 0.801 |
| Post-TH |  | ***-0.053 ± 0.020*** | ***0.009*** | ***(-0.092, -0.013)*** |  |
| TH | GFAP | ***-0.061 ± 0.024*** | ***0.012*** | ***(-0.108, -0.014)*** | 0.799 |
| Post-TH |  | ***-0.058 ± 0.026*** | ***0.024*** | ***(-0.108, -0.008)*** |  |
| TH | IL6 | -0.022 ± 0.012 | 0.071 | (-0.046, 0.002) | 0.531 |
| Post-TH |  | -0.013 ± 0.015 | 0.389 | (-0.043, 0.017) |  |
| TH | IL8 | -0.016 ± 0.011 | 0.126 | (-0.038, 0.005) | 0.962 |
| Post-TH |  | -0.016 ± 0.013 | 0.207 | (-0.041, 0.009) |  |
| TH | IL10 | ***-0.062 ± 0.018*** | ***<0.001*** | ***(-0.096, -0.027)*** | ***0.008*** |
| Post-TH |  | -0.010 ± 0.021 | 0.617 | (-0.051, 0.030) |  |
| TH | (IL6*IL8)/IL10 Index | 0.028 ± 0.020 | 0.164 | (-0.011, 0.067) | 0.070 |
| Post-TH |  | -0.016 ± 0.024 | 0.498 | (-0.064, 0.031) |  |
|  |  |  |  |  |  |
| **NRBC** |  |  |  |  |  |
| **TH Status** | **Biomarker** | **β Coefficient ± SE** | **p-value** | **95% CI** | **Inter-p-value** |
| TH | BDNF | ***-0.018 ± 0.005*** | ***0.001*** | ***(-0.029, -0.008)*** | ***0.024*** |
| Post-TH |  | ***-0.033 ± 0.006*** | ***<0.001*** | ***(-0.045, -0.022)*** |  |
| TH | VEGF | ***-0.061 ± 0.010*** | ***<0.001*** | ***(-0.080, -0.041)*** | 0.275 |
| Post-TH |  | ***-0.050 ± 0.010*** | ***<0.001*** | ***(-0.070, -0.030)*** |  |
| TH | TAU | 0.022 ± 0.015 | 0.145 | (-0.007, 0.051) | ***0.001*** |
| Post-TH |  | -0.014 ± 0.015 | 0.376 | (-0.044, 0.016) |  |
| TH | GFAP | -0.007 ± 0.020 | 0.724 | (-0.045, 0.032) | 0.312 |
| Post-TH |  | -0.016 ± 0.020 | 0.419 | (-0.055, 0.023) |  |
| TH | IL6 | ***0.033 ± 0.009*** | ***<0.001*** | ***(0.015, 0.050)*** | ***0.004*** |
| Post-TH |  | ***0.058 ± 0.010*** | ***<0.001*** | ***(0.039, 0.077)*** |  |
| TH | IL8 | ***0.026 ± 0.008*** | ***0.001*** | ***(0.010, 0.041)*** | 0.178 |
| Post-TH |  | 0.015 ± 0.008 | 0.068 | (-0.001, 0.032) |  |
| TH | IL10 | ***0.056 ± 0.013*** | ***<0.001*** | ***(0.030, 0.081)*** | 0.065 |
| Post-TH |  | ***0.032 ± 0.014*** | ***0.017*** | ***(0.006, 0.059)*** |  |
| TH | (IL6*IL8)/IL10 Index | -0.006 ± 0.015 | 0.708 | (-0.035, 0.024) | ***0.012*** |
| Post-TH |  | ***0.034 ± 0.016*** | ***0.030*** | ***(0.003, 0.066)*** |  |

| **Table S1. (Continuation). Mixed model adjusted for indicators of HI severity, time of biomarker (repeated measures) and sex** | | | | | |
| --- | --- | --- | --- | --- | --- |
| **Lactate** |  |  |  |  |  |
| **TH Status** | **Biomarker** | **β Coefficient ± SE** | **p-value** | **95% CI** | **Inter-p-value** |
| TH | BDNF | -0.002 ± 0.012 | 0.866 | (-0.026, 0.022) | 0.068 |
| Post-TH |  | -0.029 ± 0.015 | 0.056 | (-0.059, 0.001) |  |
| TH | VEGF | ***-0.090 ± 0.021*** | ***<0.001*** | ***(-0.132, -0.048)*** | 0.477 |
| Post-TH |  | ***-0.073 ± 0.026*** | ***0.005*** | ***(-0.124, -0.023)*** |  |
| TH | TAU | ***0.142 ± 0.029*** | ***<0.001*** | ***(0.084, 0.199)*** | 0.135 |
| Post-TH |  | ***0.104 ± 0.033*** | ***0.001*** | ***(0.040, 0.168)*** |  |
| TH | GFAP | ***0.135 ± 0.039*** | ***0.001*** | ***(0.059, 0.210)*** | 0.483 |
| Post-TH |  | ***0.150 ± 0.041*** | ***<0.001*** | ***(0.070, 0.231)*** |  |
| TH | IL6 | 0.031 ± 0.021 | 0.133 | (-0.009, 0.071) | 0.152 |
| Post-TH |  | ***0.064 ± 0.025*** | ***0.011*** | ***(0.015, 0.113)*** |  |
| TH | IL8 | ***0.047 ± 0.017*** | ***0.004*** | ***(0.015, 0.080)*** | 0.261 |
| Post-TH |  | 0.030 ± 0.019 | 0.120 | (-0.008, 0.067) |  |
| TH | IL10 | ***0.097 ± 0.028*** | ***<0.001*** | ***(0.042, 0.151)*** | 0.329 |
| Post-TH |  | ***0.068 ± 0.033*** | ***0.039*** | ***(0.004, 0.132)*** |  |
| TH | (IL6*IL8)/IL10 Index | -0.018 ± 0.032 | 0.565 | (-0.080, 0.044) | 0.256 |
| Post-TH |  | 0.025 ± 0.039 | 0.513 | (-0.051, 0.101) |  |
|  |  |  |  |  |  |
| **Sarnat score** |  |  |  |  |  |
| **TH Status** | **Biomarker** | **β Coefficient ± SE** | **p-value** | **95% CI** | **Inter-p-value** |
| TH | BDNF | -0.042 ± 0.080 | 0.602 | (-0.198, 0.115) | 0.350 |
| Post-TH |  | 0.061 ± 0.105 | 0.561 | (-0.145, 0.267) |  |
| TH | VEGF | -0.264 ± 0.150 | 0.078 | (-0.558, -0.030) | 0.404 |
| Post-TH |  | -0.132 ± 0.181 | 0.467 | (-0.486, 0.223) |  |
| TH | TAU | ***0.767 ± 0.186*** | ***<0.001*** | ***(0.401, 1.132)*** | 0.072 |
| Post-TH |  | ***1.082 ± 0.217*** | ***<0.001*** | ***(0.658, 1.507)*** |  |
| TH | GFAP | ***0.738 ± 0.255*** | ***0.004*** | ***(0.238, 1.237)*** | ***0.014*** |
| Post-TH |  | ***1.100 ± 0.271*** | ***<0.001*** | ***(0.569, 1.631)*** |  |
| TH | IL6 | 0.049 ± 0.134 | 0.714 | (-0.213, 0.311) | 0.432 |
| Post-TH |  | 0.166 ± 0.164 | 0.314 | (-0.157, 0.488) |  |
| TH | IL8 | 0.127 ± 0.114 | 0.265 | (-0.096, 0.350) | 0.969 |
| Post-TH |  | 0.132 ± 0.137 | 0.336 | (-0.137, 0.400) |  |
| TH | IL10 | ***0.606 ± 0.185*** | ***0.001*** | ***(0.243, 0.969)*** | 0.655 |
| Post-TH |  | ***0.514 ± 0.224*** | ***0.021*** | ***(0.076, 0.953)*** |  |
| TH | (IL6*IL8)/IL10 Index | ***-0.469 ± 0.208*** | ***0.024*** | ***(-0.876, -0.061)*** | 0.520 |
| Post-TH |  | -0.301 ± 0.260 | 0.247 | (-0.811, 0.209) |  |

| **Table S1. (Continuation).** **Mixed model adjusted for indicators of HI severity, time of biomarker (repeated measures) and sex** | | | | | |
| --- | --- | --- | --- | --- | --- |
| **PI Score** |  |  |  |  |  |
| **TH Status** | **Biomarker** | **β Coefficient ± SE** | **p-value** | **95% CI** | **Inter-p-value** |
| TH | BDNF | -0.008 ± 0.027 | 0.777 | (-0.061, 0.045) | 0.712 |
| Post-TH |  | 0.006 ± 0.033 | 0.868 | (-0.060, 0.071) |  |
| TH | VEGF | ***-0.102 ± 0.050*** | ***0.041*** | ***(-0.200, -0.004)*** | 0.911 |
| Post-TH |  | -0.096 ± 0.058 | 0.095 | (-0.210, 0.017) |  |
| TH | TAU | ***0.163 ± 0.066*** | ***0.013*** | ***(0.034, 0.293)*** | 0.745 |
| Post-TH |  | ***0.183 ± 0.074*** | ***0.013*** | ***(0.038, 0.328)*** |  |
| TH | GFAP | ***0.182 ± 0.087*** | ***0.038*** | ***(0.010, 0.353)*** | 0.665 |
| Post-TH |  | 0.160 ± 0.092 | 0.081 | (-0.020, 0.341) |  |
| TH | IL6 | 0.040 ± 0.045 | 0.374 | (-0.048, 0.127) | 0.310 |
| Post-TH |  | 0.089 ± 0.053 | 0.092 | (-0.015, 0.192) |  |
| TH | IL8 | 0.061 ± 0.038 | 0.107 | (-0.013, 0.134) | 0.565 |
| Post-TH |  | 0.037 ± 0.044 | 0.395 | (-0.049, 0.123) |  |
| TH | IL10 | ***0.198 ± 0.062*** | ***0.001*** | ***(0.077, 0.318)*** | 0.428 |
| Post-TH |  | ***0.145 ± 0.072*** | ***0.044*** | ***(0.004, 0.285)*** |  |
| TH | (IL6*IL8)/IL10 Index | -0.102 ± 0.070 | 0.144 | (-0.239, 0.035) | 0.379 |
| Post-TH |  | -0.027 ± 0.084 | 0.743 | (-0.191, 0.137) |  |

BDNF - Brain-derived neurotrophic factor

CI - Confidence Interval

GFAP - Glial fibrillary acidic protein

IL – Interleukin

NRBC - Nucleated Red Blood Cells

PI Score - Perinatal Insult Score

SE - Standard error

TH - Therapeutic hypothermia

VEGF - Vascular Endothelial Growth Factor

**Table S2. Mixed model adjusted for indicators of HI severity, time of biomarker (repeated measures), sex, and neonatal infection status**

| **Apgar at 5 min** | |  |  |  |  |
| --- | --- | --- | --- | --- | --- |
| **TH Status** | **Biomarker** | **β Coefficient ± SE** | **p-value** | **95% CI** | **Inter-p-value** |
| TH | BDNF | 0.002 ± 0.024 | 0.926 | (-0.044, 0.048) | 0.235 |
| Post-TH |  | -0.037 ± 0.032 | 0.238 | (-0.099, 0.025) |  |
| TH | VEGF | 0.052 ± 0.041 | 0.211 | (-0.029, 0.133) | 0.305 |
| Post-TH |  | 0.003 ± 0.051 | 0.951 | (-0.097, 0.103) |  |
| TH | TAU | ***-0.211 ± 0.058*** | ***<0.001*** | ***(-0.324, -0.098)*** | 0.537 |
| Post-TH |  | ***-0.177 ± 0.067*** | ***0.008*** | ***(-0.308, -0.047)*** |  |
| TH | GFAP | ***-0.273 ± 0.074*** | ***<0.001*** | ***(-0.418, -0.128)*** | ***0.042*** |
| Post-TH |  | ***-0.179 ± 0.080*** | ***0.025*** | ***(-0.336, -0.022)*** |  |
| TH | IL6 | -0.020 ± 0.039 | 0.611 | (-0.096, 0.056) | 0.643 |
| Post-TH |  | -0.040 ± 0.048 | 0.404 | (-0.135, 0.054) |  |
| TH | IL8 | -0.026 ± 0.033 | 0.429 | (-0.090, 0.038) | 0.051 |
| Post-TH |  | ***-0.098 ± 0.040*** | ***0.014*** | ***(-0.176, -0.020)*** |  |
| TH | IL10 | ***-0.101 ± 0.053*** | ***0.060*** | ***(-0.205, 0.004)*** | 0.542 |
| Post-TH |  | -0.063 ± 0.065 | 0.331 | (-0.191, 0.064) |  |
| TH | (IL6*IL8)/IL10 Index | 0.082 ± 0.061 | 0.176 | (-0.037, 0.202) | 0.155 |
| Post-TH |  | -0.025 ± 0.076 | 0.739 | (-0.175, 0.124) |  |
|  |  |  |  |  |  |
| **pH** |  |  |  |  |  |
| **TH Status** | **Biomarker** | **β Coefficient ± SE** | **p-value** | **95% CI** | **Inter-p-value** |
| TH | BDNF | 0.481 ± 0.276 | 0.081 | (-0.060, 1.021) | 0.777 |
| Post-TH |  | 0.373 ± 0.357 | 0.296 | (-0.327, 1.073) |  |
| TH | VEGF | ***0.994 ± 0.505*** | ***0.049*** | ***(0.003, 1.984)*** | 0.375 |
| Post-TH |  | ***1.476 ± 0.608*** | ***0.015*** | ***(0.284, 2.667)*** |  |
| TH | TAU | ***-1.344 ± 0.676*** | ***0.047*** | ***(-2.670, -0.019)*** | 0.624 |
| Post-TH |  | -1.036 ± 0.768 | 0.178 | (-2.541, 0.470) |  |
| TH | GFAP | -1.041 ± 0.884 | 0.239 | (-2.774, 0.692) | 0.623 |
| Post-TH |  | -0.790 ± 0.941 | 0.401 | (-2.635, 1.054) |  |
| TH | IL6 | ***-1.009 ± 0.449*** | ***0.025*** | ***(-1.889, -0.128)*** | 0.501 |
| Post-TH |  | ***-1.354 ± 0.550*** | ***0.014*** | ***(-2.432, -0.276)*** |  |
| TH | IL8 | ***-0.867 ± 0.362*** | ***0.017*** | ***(-1.577, -0.157)*** | 0.100 |
| Post-TH |  | -0.187 ± 0.440 | 0.670 | (-1.049, 0.675) |  |
| TH | IL10 | ***-1.305 ± 0.610*** | ***0.032*** | ***(-2.501, -0.109)*** | 0.296 |
| Post-TH |  | -0.591 ± 0.736 | 0.422 | (-2.034, 0.852) |  |
| TH | (IL6*IL8)/IL10 Index | -0.502 ± 0.687 | 0.465 | (-1.849, 0.845) | 0.660 |
| Post-TH |  | -0.884 ± 0.859 | 0.303 | (-2.568, 0.800) |  |

| **Table S2. (Continuation).** **Mixed model adjusted for indicators of HI severity, time of biomarker (repeated measures), sex, and neonatal infection status** | | | | | |
| --- | --- | --- | --- | --- | --- |
| **Base Excess** | |  |  |  |  |
| **TH Status** | **Biomarker** | **β Coefficient ± SE** | **p-value** | **95% CI** | **Inter-p-value** |
| TH | BDNF | 0.008 ± 0.007 | 0.269 | (-0.006, 0.023) | 0.448 |
| Post-TH |  | <0.001 ± 0.010 | 0.977 | (-0.019, 0.020) |  |
| TH | VEGF | ***0.045 ± 0.013*** | ***0.001*** | ***(0.019, 0.071)*** | 0.288 |
| Post-TH |  | 0.029 ± 0.016 | 0.080 | (-0.003, 0.061) |  |
| TH | TAU | ***-0.058 ± 0.018*** | ***0.001*** | ***(-0.093, -0.024)*** | 0.811 |
| Post-TH |  | ***-0.054 ± 0.020*** | ***0.007*** | ***(-0.094, -0.015)*** |  |
| TH | GFAP | ***-0.062 ± 0.024*** | ***0.010*** | ***(-0.110, -0.015)*** | 0.806 |
| Post-TH |  | ***-0.059 ± 0.026*** | ***0.022*** | ***(-0.109, -0.009)*** |  |
| TH | IL6 | -0.020 ± 0.012 | 0.105 | (-0.043, 0.004) | 0.489 |
| Post-TH |  | -0.010 ± 0.015 | 0.518 | (-0.040, 0.020) |  |
| TH | IL8 | -0.014 ± 0.011 | 0.178 | (-0.035, 0.007) | 0.927 |
| Post-TH |  | -0.013 ± 0.013 | 0.288 | (-0.038, 0.011) |  |
| TH | IL10 | ***-0.058 ± 0.018*** | ***0.001*** | ***(-0.093, -0.024)*** | ***0.007*** |
| Post-TH |  | -0.006 ± 0.021 | 0.758 | (-0.047, 0.034) |  |
| TH | (IL6*IL8)/IL10 Index | 0.030 ± 0.020 | 0.139 | (-0.010, 0.069) | 0.073 |
| Post-TH |  | -0.014 ± 0.024 | 0.562 | (-0.062, 0.033) |  |
|  |  |  |  |  |  |
| **NRBC** |  |  |  |  |  |
| **TH Status** | **Biomarker** | **β Coefficient ± SE** | **p-value** | **95% CI** | **Inter-p-value** |
| TH | BDNF | ***-0.019 ± 0.005*** | ***<0.001*** | ***(-0.030, -0.008)*** | ***0.024*** |
| Post-TH |  | ***-0.034 ± 0.006*** | ***<0.001*** | ***(-0.045, -0.022)*** |  |
| TH | VEGF | ***-0.059 ± 0.010*** | ***<0.001*** | ***(-0.077, -0.040)*** | 0.270 |
| Post-TH |  | ***-0.048 ± 0.010*** | ***<0.001*** | ***(-0.067, -0.029)*** |  |
| TH | TAU | 0.020 ± 0.015 | 0.180 | (-0.009, 0.049) | ***0.001*** |
| Post-TH |  | -0.015 ± 0.015 | 0.322 | (-0.045, 0.015) |  |
| TH | GFAP | -0.005 ± 0.020 | 0.805 | (-0.043, 0.034) | 0.313 |
| Post-TH |  | -0.014 ± 0.020 | 0.483 | (-0.053, 0.025) |  |
| TH | IL6 | ***0.032 ± 0.009*** | ***<0.001*** | ***(0.014, 0.050)*** | ***0.004*** |
| Post-TH |  | ***0.057 ± 0.010*** | ***<0.001*** | ***(0.039, 0.076)*** |  |
| TH | IL8 | ***0.024 ± 0.008*** | ***0.003*** | ***(0.008, 0.039)*** | 0.177 |
| Post-TH |  | 0.013 ± 0.008 | 0.107 | (-0.003, 0.029) |  |
| TH | IL10 | ***0.051 ± 0.012*** | ***<0.001*** | ***(0.027, 0.075)*** | 0.063 |
| Post-TH |  | ***0.028 ± 0.013*** | ***0.036*** | ***(0.002, 0.053)*** |  |
| TH | (IL6*IL8)/IL10 Index | -0.004 ± 0.015 | 0.790 | (-0.033, 0.025) | ***0.012*** |
| Post-TH |  | ***0.036 ± 0.016*** | ***0.023*** | ***(0.005, 0.067)*** |  |

| **Table S2. (Continuation).** **Mixed model adjusted for indicators of HI severity, time of biomarker (repeated measures), sex, and neonatal infection status** | | | | | |
| --- | --- | --- | --- | --- | --- |
| **Lactate** |  |  |  |  |  |
| **TH Status** | **Biomarker** | **β Coefficient ± SE** | **p-value** | **95% CI** | **Inter-p-value** |
| TH | BDNF | -0.005 ± 0.012 | 0.715 | (-0.029, 0.020) | 0.063 |
| Post-TH |  | ***-0.032 ± 0.015*** | ***0.037*** | ***(-0.062, -0.002)*** |  |
| TH | VEGF | ***-0.084 ± 0.021*** | ***<0.001*** | ***(-0.125, -0.042)*** | 0.447 |
| Post-TH |  | ***-0.066 ± 0.026*** | ***0.011*** | ***(-0.117, -0.015)*** |  |
| TH | TAU | ***0.143 ± 0.029*** | ***<0.001*** | ***(0.085, 0.200)*** | 0.138 |
| Post-TH |  | ***0.106 ± 0.033*** | ***0.001*** | ***(0.041, 0.170)*** |  |
| TH | GFAP | ***0.135 ± 0.039*** | ***0.001*** | ***(0.058, 0.211)*** | 0.483 |
| Post-TH |  | ***0.150 ± 0.041*** | ***<0.001*** | ***(0.069, 0.231)*** |  |
| TH | IL6 | 0.025 ± 0.021 | 0.226 | (-0.015, 0.065) | 0.166 |
| Post-TH |  | ***0.057 ± 0.025*** | ***0.024*** | ***(0.008, 0.106)*** |  |
| TH | IL8 | ***0.042 ± 0.017*** | ***0.011*** | ***(0.010, 0.075)*** | 0.244 |
| Post-TH |  | 0.024 ± 0.019 | 0.212 | (-0.014, 0.061) |  |
| TH | IL10 | ***0.090 ± 0.028*** | ***0.001*** | ***(0.035, 0.144)*** | 0.311 |
| Post-TH |  | 0.060 ± 0.033 | 0.070 | (-0.005, 0.124) |  |
| TH | (IL6*IL8)/IL10 Index | -0.023 ± 0.032 | 0.471 | (-0.085, 0.039) | 0.267 |
| Post-TH |  | 0.020 ± 0.039 | 0.615 | (-0.057, 0.096) |  |
|  |  |  |  |  |  |
| **Sarnat score** |  |  |  |  |  |
| **TH Status** | **Biomarker** | **β Coefficient ± SE** | **p-value** | **95% CI** | **Inter-p-value** |
| TH | BDNF | -0.041 ± 0.080 | 0.611 | (-0.197, 0.116) | 0.351 |
| Post-TH |  | 0.062 ± 0.105 | 0.556 | (-0.144, 0.268) |  |
| TH | VEGF | -0.246 ± 0.148 | 0.096 | (-0.536, 0.043) | 0.412 |
| Post-TH |  | -0.116 ± 0.179 | 0.517 | (-0.467, 0.235) |  |
| TH | TAU | ***0.773 ± 0.187*** | ***<0.001*** | ***(0.407, 1.139)*** | 0.071 |
| Post-TH |  | ***1.088 ± 0.217*** | ***<0.001*** | ***(0.663, 1.513)*** |  |
| TH | GFAP | ***0.744 ± 0.255*** | ***0.004*** | ***(0.244, 1.243)*** | ***0.014*** |
| Post-TH |  | ***1.105 ± 0.271*** | ***<0.001*** | ***(0.574, 1.637)*** |  |
| TH | IL6 | 0.034 ± 0.132 | 0.795 | (-0.225, 0.294) | 0.433 |
| Post-TH |  | 0.150 ± 0.163 | 0.356 | (-0.169, 0.470) |  |
| TH | IL8 | 0.107 ± 0.113 | 0.342 | (-0.114, 0.328) | 0.972 |
| Post-TH |  | 0.112 ± 0.136 | 0.411 | (-0.155, 0.378) |  |
| TH | IL10 | ***0.577 ± 0.184*** | ***0.002*** | ***(0.216, 0.939)*** | 0.657 |
| Post-TH |  | ***0.487 ± 0.222*** | ***0.029*** | ***(0.051, 0.922)*** |  |
| TH | (IL6*IL8)/IL10 Index | ***-0.485 ± 0.208*** | ***0.020*** | ***(-0.892, -0.078)*** | 0.519 |
| Post-TH |  | -0.317 ± 0.260 | 0.223 | (-0.826, 0.192) |  |

| **Table S2. (Continuation).** **Mixed model adjusted for indicators of HI severity, time of biomarker (repeated measures), sex, and neonatal infection status** | | | | | |
| --- | --- | --- | --- | --- | --- |
| **PI Score** |  |  |  |  |  |
| **TH Status** | **Biomarker** | **β Coefficient ± SE** | **p-value** | **95% CI** | **Inter-p-value** |
| TH | BDNF | -0.007 ± 0.027 | 0.787 | (-0.060, 0.046) | 0.709 |
| Post-TH |  | 0.006 ± 0.034 | 0.857 | (-0.060, 0.072) |  |
| TH | VEGF | ***-0.096 ± 0.049*** | ***0.051*** | ***(-0.193, 0.001)*** | 0.885 |
| Post-TH |  | -0.089 ± 0.057 | 0.122 | (-0.201, 0.024) |  |
| TH | TAU | ***0.165 ± 0.066*** | ***0.013*** | ***(0.035, 0.294)*** | 0.741 |
| Post-TH |  | ***0.184 ± 0.074*** | ***0.013*** | ***(0.039, 0.330)*** |  |
| TH | GFAP | ***0.183 ± 0.088*** | ***0.036*** | ***(0.012, 0.355)*** | 0.667 |
| Post-TH |  | 0.162 ± 0.092 | 0.079 | (-0.019, 0.342) |  |
| TH | IL6 | 0.035 ± 0.044 | 0.431 | (-0.052, 0.122) | 0.327 |
| Post-TH |  | 0.082 ± 0.052 | 0.117 | (-0.020, 0.184) |  |
| TH | IL8 | 0.054 ± 0.037 | 0.150 | (-0.019, 0.127) | 0.547 |
| Post-TH |  | 0.029 ± 0.043 | 0.502 | (-0.056, 0.114) |  |
| TH | IL10 | ***0.187 ± 0.061*** | ***0.002*** | ***(0.067, 0.307)*** | 0.415 |
| Post-TH |  | ***0.133 ± 0.071*** | ***0.063*** | ***(-0.007, 0.273)*** |  |
| TH | (IL6*IL8)/IL10 Index | -0.108 ± 0.070 | 0.125 | (-0.245, 0.030) | 0.384 |
| Post-TH |  | -0.034 ± 0.084 | 0.689 | (-0.198, 0.131) |  |

BDNF - Brain-derived neurotrophic factor

CI - Confidence Interval

GFAP - Glial fibrillary acidic protein

IL – Interleukin

NRBC - Nucleated Red Blood Cells

PI Score - Perinatal Insult Score

SE - Standard error

TH - Therapeutic hypothermia

VEGF - Vascular Endothelial Growth Factor
